# Facile access to pyridinium-based bent aromatic amphiphiles: nonionic surface modification of nanocarbons in water

**DOI:** 10.3762/bjoc.20.5

**Published:** 2024-01-08

**Authors:** Lorenzo Catti, Shinji Aoyama, Michito Yoshizawa

**Affiliations:** 1 Laboratory for Chemistry and Life Science, Institute of Innovative Research, Tokyo Institute of Technology, 4259 Nagatsuta, Midori-ku, Yokohama 226-8503, Japan

**Keywords:** aromatic micelle, nanocarbon, nonionic surface modification, pyridinium, water-solubilization

## Abstract

Efficient water-solubilization of nanocarbons is desirable for both their biological and material applications, but so far has mainly relied on covalent modifications or amphiphiles featuring ionic side-chains. Here, we report a facile 2–4-step synthesis of pyridinium-based, bent aromatic amphiphiles with modular *nonionic side-chains* (i.e., CH_3_ and CH_2_CH_2_(OCH_2_CH_2_)_2_–Y (Y = OCH_3_, OH, and imidazole)). The new amphiphiles quantitatively self-assemble into ≈2 nm-sized aromatic micelles in water independent of the side-chain. Importantly, efficient water-solubilization and *nonionic* surface modification of various nanocarbons (e.g., fullerene C_60_, carbon nanotubes, and graphene nanoplatelets) are achieved through noncovalent encircling with the bent amphiphiles. The resultant imidazole-modified nanocarbons display a pH-responsive surface charge, as evidenced by NMR and zeta-potential measurements. In addition, solubilization of a nitrogen-doped nanocarbon (i.e., graphitic carbon nitride) in the form of 10–30 nm-sized stacks is also demonstrated using the present amphiphiles.

## Introduction

**Figure 1:**
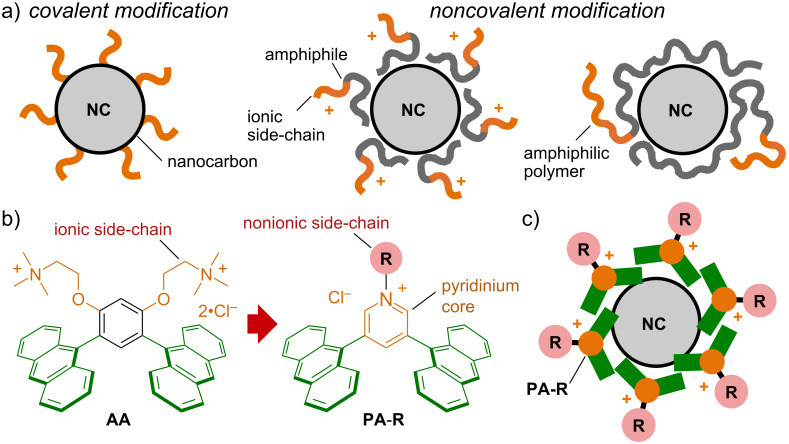
a) Previous methods for the water-solubilization and modification of nanocarbons (NCs). b) Bent aromatic amphiphiles **AA** and **PA**-**R** (R = CH_3_ and CH_2_CH_2_(OCH_2_CH_2_)_2_–Y (Y = OCH_3_, OH, and imidazole)). c) Nonionic surface modification of NCs via noncovalent encircling with **PA**-**R**s.

Nanocarbons, such as fullerenes, graphenes, and carbon nanotubes, are continuing to attract global attention due to their unique chemical and physical properties [1–2]. Facile water-solubilization and modifications of nanocarbons with various sizes and shapes are desirable for both their biological and materials applications, but so far have mainly relied on i) covalent modifications, leading to irreversible property changes (Figure 1a, left) [3–4] and noncovalent wrapping with ii) linear/planar amphiphiles bearing ionic side-chains [5–6] or iii) amphiphilic/polar polymers, displaying low to moderate interactions and debundling efficiency (Figure 1a, center and right) [7–11]. The development of new amphiphiles with large polyaromatic panels for strong aromatic–aromatic interactions and nonionic side-chains on the other hand would grant access to water-soluble nanocarbons with tunable surface properties, which could expand noncovalent nanocarbon chemistry and technology.

We here report pyridinium-based, bent aromatic amphiphiles **PA**-**R**, featuring a pyridinium salt (Py-R^+^**·**Cl^−^) as the key motif, capable of providing both a cationic hydrophilic hinge and a variety of nonionic side-chains (i.e., CH_3_ and CH_2_CH_2_(OCH_2_CH_2_)_2_–Y (Y = OCH_3_, OH, and imidazole); Figure 1b). The present amphiphile is originated from bent aromatic amphiphile **AA** [12–13], composed of two anthracene panels linked by a *m*-phenylene spacer with two cationic side-chains, which assembles into an aqueous ≈2 nm-sized aromatic micelle with broad host functions [14–21]. Replacement of the *m*-phenylene unit with an *N*-functionalized pyridinium core reduces the synthetic steps from 6 to 2–4, while at the same time enabling the use of various nonionic side-chains. Amphiphiles **PA**-**R** quantitatively self-assemble into aromatic micelles (**PA**-**R**)*_n_*, with outer diameters of ≈2 nm, independent of the side-chain present. Importantly, efficient water-solubilization and nonionic surface modification of nanocarbons (i.e., fullerene C_60_ (**C_60_**), single/multi-walled carbon nanotubes (***s*/*m***-**CNT**), and graphene nanoplatelets (**GN**)) can be achieved through noncovalent encircling with the present pyridinium-based amphiphiles (Figure 1c). The noncovalent modification of **C_60_** with multiple imidazole side-chains is found to yield a pH-responsive surface charge, as evidenced by NMR and zeta-potential measurements. A nitrogen-doped nanocarbon, i.e., graphitic carbon nitride (**g**-**C_3_N_4_**), is likewise solubilized by **PA**-**R** in water and subsequently deposited onto a cellulose filter using a simple filtration protocol.

## Results and Discussion

### Synthesis of pyridinium-based amphiphiles

**Figure 2:**
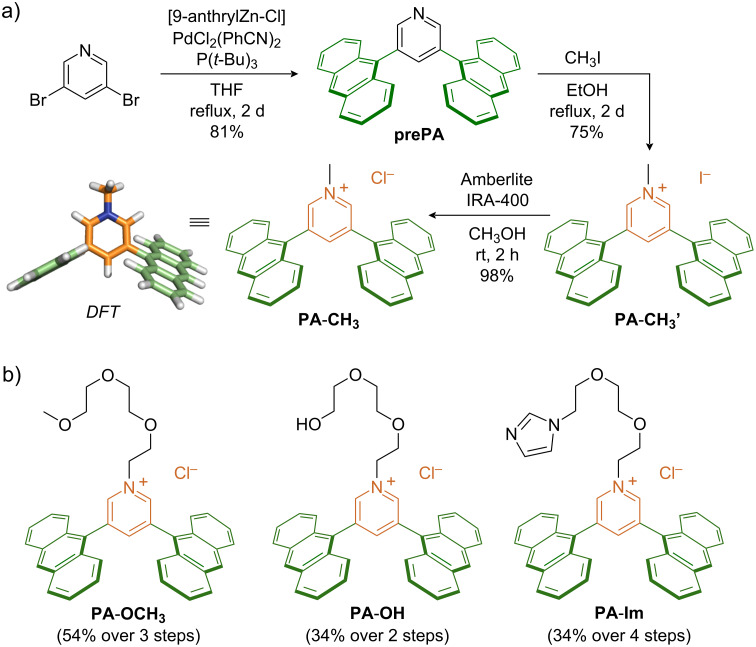
a) Synthetic route toward **prePA** and **PA**-**CH_3_**, including the optimized structure (DFT) of **PA**-**CH_3_**. b) Structures of **PA**-**OCH_3_**, **PA**-**OH**, and **PA**-**Im**.

Synthesis of the pyridinium-based amphiphiles **PA**-**R** was achieved in 2–4 steps starting from commercially available 3,5-dibromopyridine. Negishi cross-coupling with 9-anthrylzinc chloride in the presence of PdCl_2_(PhCN)_2_/P(*t*-Bu)_3_ as catalyst afforded the common precursor 3,5-dianthrylpyridine (**prePA**), a simple yet novel bent building block, in 81% yield. For the synthesis of the methyl derivative, **prePA** was *N*-alkylated with excess methyl iodide yielding **PA**-**CH_3_’**, followed by ion exchange using an exchange resin to provide **PA**-**CH_3_** as a yellow solid (74% yield over 2 steps; Figure 2a). Despite its large aromatic framework with a monocationic core, **PA**-**CH_3_** was found to be soluble in water up to ≈0.9 mM. Using similar procedures, amphiphiles **PA**-**OCH_3_** and **PA**-**OH** were synthesized by reacting **prePA** under neat conditions with 1-(2-bromoethoxy)-2-(2-methoxyethoxy)ethane (67% yield over 2 steps after ion-exchange) and 2-[2-(2-chloroethoxy)ethoxy]ethanol (42% yield), respectively (Figure 2b). Imidazole-functionalized amphiphile **PA**-**Im** was obtained by first reacting **prePA** with 1,2-bis(iodoethoxy)ethane to yield a reactive iodo-derivative, which was subsequently quaternized with imidazole and converted to the chloride salt (42% yield over 3 steps). All three CH_2_CH_2_(OCH_2_CH_2_)_2_-containing amphiphiles were found to display good-to-high water-solubilities. It is noteworthy that an **AA** derivative featuring three nonionic –O(CH_2_CH_2_O)_2_CH_3_ side-chains on the phenylene spacer is insoluble in water (see Figure S45 in Supporting Information File 1), emphasizing the importance of the hydrophilic pyridinium core for the observed water-solubility.

### Formation and structure of aromatic micelles

**Figure 3:**
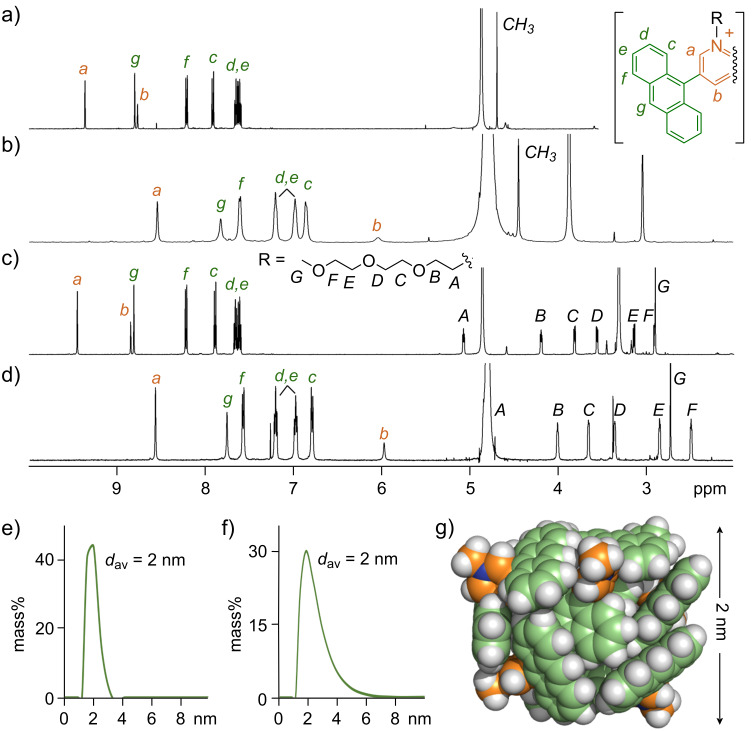
^1^H NMR spectra (500 MHz, rt, 0.5 mM and 1.0 mM based on **PA**-**CH_3_** and **PA**-**OCH_3_**, respectively, TMS in CDCl_3_ as external standard) of a) **PA**-**CH_3_** in CD_3_OD, b) (**PA**-**CH_3_**)*_n_* in D_2_O, c) **PA**-**OCH_3_** in CD_3_OD, and d) (**PA**-**OCH_3_**)*_n_* in D_2_O. DLS charts (rt, H_2_O, 0.5 mM and 1.0 mM based on **PA**-**CH_3_** and **PA**-**OCH_3_**, respectively) of e) (**PA**-**CH_3_**)*_n_* and f) (**PA**-**OCH_3_**)*_n_*. g) Optimized structure (MM) of (**PA**-**CH_3_**)_6_.

Aromatic micelle (**PA**-**CH_3_**)*_n_* was facilely generated via dissolution of **PA**-**CH_3_** (2.1 mg, 4.3 μmol) in water (8.6 mL). The ^1^H NMR spectrum of **PA**-**CH_3_** in D_2_O showed significantly upfield-shifted signals (Δδ = −2.73 ppm for *H*_b_) compared to the spectrum in CD_3_OD, in a manner similar to **AA** [12–13], indicating self-assembly via the hydrophobic effect and π-stacking interactions (Figure 3a,b). The self-assembly in water was further supported by UV–visible analysis, displaying slight red-shifts of the anthracene absorption bands relative to the spectrum in methanol (Δλ_max_ = +3; Figure S36 in Supporting Information File 1). The formation of spherical particles with narrow size distribution and an average diameter of ≈2 nm was confirmed by DLS and DOSY NMR measurements (Figure 3e and Figure S27 in Supporting Information File 1). Based on molecular modeling, these data suggested the self-assembly of in average six **PA**-**CH_3_** amphiphiles into a small aromatic micelle (Figure 3g). In a similar way, the generation of micelles (**PA**-**OCH_3_**)*_n_*, (**PA**-**OH**)*_n_*, and (**PA**-**Im**)*_n_* was confirmed via NMR, UV–visible, and DLS analyses upon dissolution of the corresponding amphiphiles in water (Figure 3c,d,f and Figure S30–S36 in Supporting Information File 1). It is noteworthy that all four amphiphiles formed similar sized micelles (*d*_av_ ≈ 2 nm), independent of the attached side-chain.

NMR dilution experiments with **PA**-**CH_3_** and **PA**-**OCH_3_** indicated that their critical micelle concentrations (CMCs) are below 0.1 mM (Figures S28 and S32, Supporting Information File 1), which is around 10 times lower than that of **AA** [12–13]. The increased stability against dilution likely arises from reduced electrostatic repulsion and increased anthracene-based π-stacking interactions due to the absence of *o*-alkoxy groups. Aromatic micelle (**PA**-**CH_3_**)*_n_* was furthermore found to be stable for at least six days at room temperature in the dark (see Figure S37 in Supporting Information File 1).

### Noncovalent encircling of various nanocarbons in water

**Figure 4:**
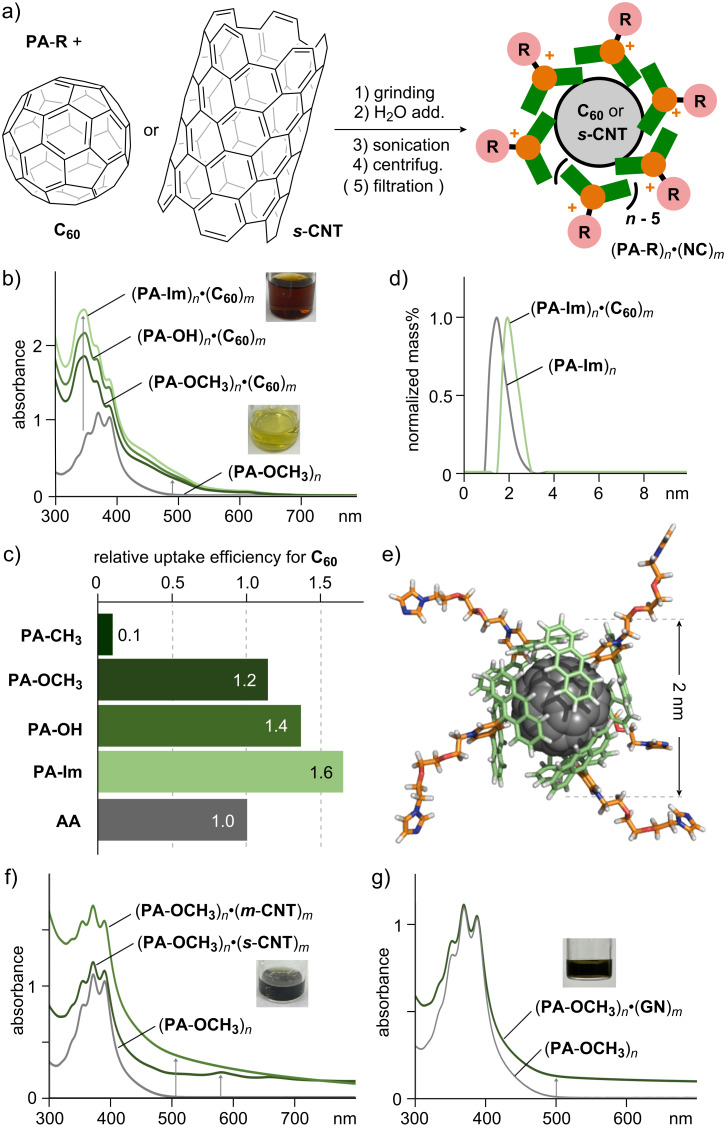
a) General protocol for the noncovalent encircling of **C_60_** and ***s***-**CNT** by **PA**-**R**. b) UV–visible spectra (H_2_O, rt, 1.0 mM based on **PA**-**R**) of (**PA**-**OCH_3_**)*_n_*, (**PA**-**OCH_3_**)*_n_***·**(**C_60_**)*_m_*, (**PA**-**OH**)*_n_***·**(**C_60_**)*_m_*, and (**PA**-**Im**)*_n_***·**(**C_60_**)*_m_*. c) Relative uptake efficiencies (λ_det_ = 525 nm) of **C_60_** by **PA**-**R** and **AA**. d) DLS charts (rt, H_2_O, 1.0 mM based on **PA**-**Im**) of (**PA**-**Im**)*_n_***·**(**C_60_**)*_m_* and (**PA**-**Im**)*_n_*. e) Optimized structure (MM) of (**PA**-**Im**)_5_**·C_60_**. UV–visible spectra (H_2_O, rt, 1.0 mM based on **PA**-**OCH_3_**) of f) (**PA**-**OCH_3_**)*_n_* and (**PA**-**OCH_3_**)*_n_***·**(***s***-**CNT** or ***m***-**CNT**)*_m_*, and g) (**PA**-**OCH_3_**)*_n_* and (**PA**-**OCH_3_**)*_n_***·**(**GN**)*_m_*.

By employing a simple grinding–sonication protocol, various nanocarbons could be efficiently solubilized in water via noncovalent encircling by newly synthesized amphiphiles **PA**-**R** [22]. A mixture of solid **PA**-**OCH_3_** (1.7 mg, 2.7 μmol) and water-insoluble **C_60_** (1.4 mg, 1.9 μmol) was manually ground for 3 min using an agate mortar and pestle (Figure 4a) [23]. Following addition of H_2_O (2.7 mL), the suspension was sonicated with a probe sonicator (40 kHz, 150 W, 10 min), centrifuged (16,000*g*, 10 min), and then filtrated (200 nm pore-size membrane filter) to yield a clear brown solution containing host–guest composite (**PA**-**OCH_3_**)*_n_***·**(**C_60_**)*_m_*. The UV–visible analysis clearly showed new guest-derived absorption bands around 340 nm and 420–650 nm, confirming the solubilization of **C_60_** in water (Figure 4b). Similar host–guest composites were also obtained using **PA**-**CH_3_**, **PA**-**OH**, and **PA**-**Im** under the same conditions. It is noteworthy that the **C_60_**-solubilization efficiency varied depending on the employed **PA**-**R** side-chain, likely due to the difference in water-solubility of the corresponding amphiphiles. The efficiency was found to be in the order of **PA**-**Im** > **PA**-**OH** > **PA**-**OCH_3_** >> **PA**-**CH_3_**, as judged by the guest absorbance at 525 nm (Figure 4c). Importantly, **PA**-**OCH_3_**, **PA**-**OH**, and **PA**-**Im** displayed an improved **C_60_**-solubilization efficiency (up to 1.6-fold), compared to previous amphiphile **AA** bearing ionic side-chains [17]. The improvement presumably stems from the increased flexibility of the anthracene panels and decreased electrostatic repulsion between the cationic moieties, allowing for tighter and more adaptable aromatic–aromatic stacking interactions. The aliphatic amphiphile sodium dodecyl sulfate (SDS) showed a 10-times lower efficiency compared to **PA**-**Im** under the same conditions (Figure S38 in Supporting Information File 1). The concentration of **C_60_** in the solution of (**PA**-**Im**)*_n_***·**(**C_60_**)*_m_* was roughly estimated to be 0.1 mg mL^−1^. The DLS measurement of (**PA**-**Im**)*_n_***·**(**C_60_**)*_m_* displayed an average particle diameter of ≈2 nm, which, in combination with molecular modeling, indicated a noncovalent surface functionalization of a single **C_60_** molecule by five **PA**-**Im** amphiphiles (Figure 4d,e).

Besides spherical **C_60_**, planar **GN** (2–10 nm thick, 5 μm wide) and tubular ***s***-**CNT** and ***m***-**CNT** (0.7–0.9 and 9–11 nm thick, ≥0.7 and 3–6 μm long, respectively) were also solubilized in water using **PA**-**OCH_3_** as a representative **PA**-**R** amphiphile, in a similar way as described above (Figure 4f,g) [23]. Due to the large size of these nanocarbons, the resultant host–guest composites were subjected to UV–visible analysis without further filtration following centrifugation (16,000*g*, ≈10 min). The obtained UV–visible spectra of the blackish, clear solutions of (**PA**-**OCH_3_**)*_n_***·**(**GN**)*_m_*, (**PA**-**OCH_3_**)*_n_***·**(***s***-**CNT**)*_m_*, and (**PA**-**OCH_3_**)*_n_***·**(***m***-**CNT**)*_m_* showed broad, featureless absorption bands, with a characteristic small peak at ≈580 nm for the E_22_ transition only in the case of the ***s***-**CNT** host–guest composite (Figure 4f,g) [24]. The concentration of **GN** in the aqueous solution of (**PA**-**OCH_3_**)*_n_***·**(**GN**)*_m_* was estimated to be ≈0.03 mg mL^−1^. Amphiphile **PA**-**OCH_3_** thus enabled the efficient water-solubilization of nanocarbons independent of their size and shape (i.e., spherical, planar, and tubular objects).

### Electrostatic surface properties of hosts and host–guest composites

Zeta potential (ZP) measurements were employed to further study the electrostatic surface properties of the aromatic micelles (**PA**-**R**)*_n_* and their host–guest composites including nanocarbons in water. The ZP of self-assembled nanoparticles evaluates their structural stability in solution against aggregation through electrostatic repulsion [25]. Solutions of the micelles in Milli-Q water (0.5 mM based on **PA**-**R**) gave ZPs significantly varying with the attached side-chains. Aqueous solutions of (**PA**-**CH_3_**)*_n_*, (**PA**-**OCH_3_**)*_n_*, and (**PA**-**OH**)*_n_* provided ZPs of 7.3 mV, 18.8 mV, and 20.3 mV, respectively, suggesting an increase in stability upon introduction of the long hydrophilic side-chains (Table 1). As expected, previous aromatic micelle (**AA**)*_n_* (1.0 mM based on **AA**) featuring multiple ionic side-chains showed a significantly higher ZP of 48.9 mV.

**Table 1:** Zeta potentials (ZP) of aromatic micelles (**PA**-**R**)*_n_* and (**AA**)*_n_* [23].

aromatic micelle	ZP [mV]

(**PA**-**CH_3_**)*_n_*	7.3
(**PA**-**OCH_3_**)*_n_*	18.8
(**PA**-**OH**)*_n_*	20.3
(**PA**-**Im**)*_n_*	41.7
(**AA**)*_n_*	48.9

**Figure 5:**
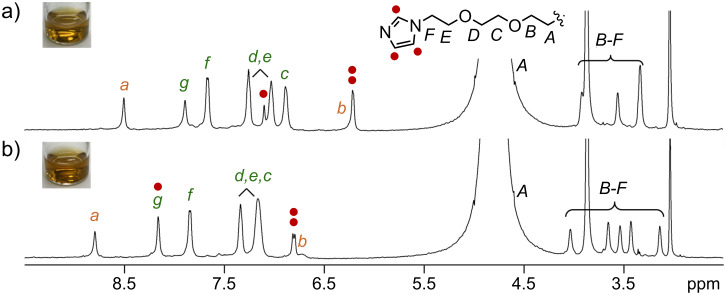
^1^H NMR spectra (500 MHz, D_2_O, rt, 0.5 mM based on **PA**-**Im**) of (**PA**-**Im**)*_n_***·**(**C_60_**)*_m_* a) before and b) after addition of 40 equiv HCl.

The ZP of (**PA**-**OCH_3_**)*_n_* largely increased upon encapsulation of **C_60_** (46.7 mV) and ***s***-**CNT** (43.2 mV), due to the stabilization of the host–guest composites through the template effect of the hydrophobic nanocarbon guests (Table 2). Moreover, aromatic micelle (**PA**-**Im**)*_n_* and its host–guest composite (**PA**-**Im**)*_n_***·**(**C_60_**)*_m_* were anticipated to provide pH-dependent ZPs via imidazole-based protonation/deprotonation. Micelle (**PA**-**Im**)*_n_* showed a significantly higher ZP (41.7 mV) compared to the other derivatives in neutral water, owing to the basic imidazole groups (Table 1). The protonation behavior of the imidazole groups on (**PA**-**Im**)*_n_***·**(**C_60_**)*_m_* in D_2_O (0.5 mM based on **PA**-**Im**) was monitored by ^1^H NMR spectroscopy, showing significant down-field shifts of the imidazole ring protons upon addition of HCl (40 equiv based on **PA**-**Im**; Figure 5a,b). These properties enabled a pH-responsive surface charge of its host–guest composite (Table 2). Accordingly, (**PA**-**Im**)*_n_***·**(**C_60_**)*_m_* displayed a ZP of 60.3 mV at pH 2.8 contrary to 52.8 mV at pH 6.8 (ΔZP = +7.5 mV) [26]. Control experiments using (**PA**-**OCH_3_**)*_n_***·**(**C_60_**)*_m_* showed only a minor change in the ZP (ΔZP = +1.0 mV) under similar conditions, confirming the importance of the noncovalent imidazole functionalities [27].

**Table 2:** Zeta potentials of (**PA**-**OCH_3_**)*_n_***·**(**C_60_** or ***s*-CNT**)*_m_* and (**PA**-**Im**)*_n_***·**(**C_60_**)*_m_* under neutral/acidic conditions [23].

host–guest composite	ZP [mV]	

	neutral	acidic

(**PA**-**OCH_3_**)*_n_***·**(**C_60_**)*_m_*	46.7	47.7
(**PA**-**OCH_3_**)*_n_***·**(***s*-CNT**)*_m_*	43.2	n.d.
(**PA**-**Im**)*_n_***·**(**C_60_**)*_m_*	52.8	60.3

### Solubilization and AFM analysis of a nitrogen-doped nanocarbon

**Figure 6:**
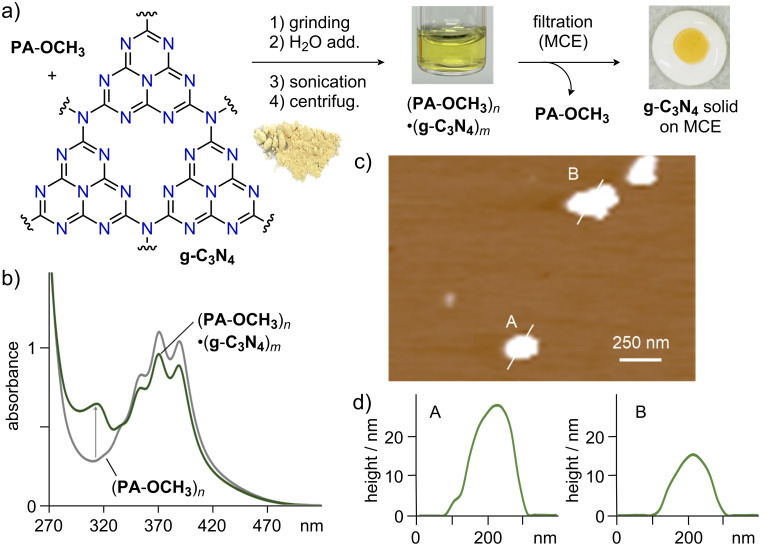
a) Protocol for the noncovalent encircling of **g**-**C_3_N_4_** by **PA**-**OCH_3_** and subsequent deposit of **g**-**C_3_N_4_** on a filter. b) UV–visible spectra (H_2_O, rt, 1.0 mM based on **PA**-**OCH_3_**) of (**PA**-**OCH_3_**)*_n_* and (**PA**-**OCH_3_**)*_n_***·**(**g**-**C_3_N_4_**)*_m_*. c) AFM image (dry, rt, mica) of (**PA**-**OCH_3_**)*_n_***·**(**g**-**C_3_N_4_**)*_m_* and d) its selected size profiles.

Finally, water-solubilization and aqueous processing of nitrogen-doped nanocarbon **g**-**C_3_N_4_** was achieved using aromatic micelle (**PA**-**OCH_3_**)*_n_* (Figure 6a). The multiple N-atoms bestow **g**-**C_3_N_4_** with unique properties that are responsible for its widespread use in catalysis [28]. Subjecting yellow solid **PA**-**OCH_3_** (1.2 mg, 1.9 μmol) and pale yellow solid **g**-**C_3_N_4_** (1.0 mg) to the grinding (3 min) and sonication (30 min) protocol provided a clear yellow aqueous solution of (**PA**-**OCH_3_**)*_n_***·**(**g**-**C_3_N_4_**)*_m_*. The formation of the host–guest structure was confirmed by UV–visible analysis, which showed a new absorption band around 312 nm (Figure 6b). This result showcased the ability of amphiphile **PA**-**OCH_3_** to solubilize not only conventional nanocarbons but also heteroatom-containing ones. To obtain direct structural information of the encircled (**g**-**C_3_N_4_**)*_m_*, dry-state AFM measurements (on mica) were conducted using an aqueous solution of (**PA**-**OCH_3_**)*_n_***·**(**g**-**C_3_N_4_**)*_m_*. The obtained AFM images showed large sheets with thicknesses of 10–30 nm and widths of 150–300 nm (Figure 6c,d), indicating solubilization of (**g**-**C_3_N_4_**)*_m_* as 20–60 stacks.

The encircled **g**-**C_3_N_4_**-based host–guest composite was subsequently deposited onto a mixed cellulose ester (MCE) filter (50 nm pore-size) using a simple filtration protocol (Figure 6a, right). Filtration of the aqueous solution of (**PA**-**OCH_3_**)*_n_***·**(**g**-**C_3_N_4_**)*_m_* and washing with water to remove **PA**-**OCH_3_** generated a yellow solid (**g**-**C_3_N_4_**)*_m_* (*d* ≈ 0.5 cm) on the filter [29]. We thus successfully demonstrated the application of (**PA**-**OCH_3_**)*_n_* toward aqueous processing of carbon/nitrogen-rich materials.

## Conclusion

We have developed new pyridinium-based bent amphiphiles **PA**-**R** that can be facilely accessed from simple yet novel building block 3,5-dianthrylpyridine in 1–3 steps. The amphiphiles quantitatively self-assembled into ≈2 nm-sized aromatic micelles (**PA**-**R**)*_n_* via the hydrophobic effect and π-stacking interactions, and displayed high stability against dilution (CMC < 0.1 mM). The molecular design allowed installation of various nonionic side-chains (i.e., CH_3_ and CH_2_CH_2_(OCH_2_CH_2_)_2_–Y (Y = OCH_3_, OH, and imidazole)) via simple *N*-alkylation, enabling the nonionic surface modification of nanocarbons via their encircling by the present amphiphiles in water. For example, utilizing the imidazole-functionalized amphiphile, an aqueous fullerene-based host–guest composite with pH-responsive surface charge was generated. In addition, water-solubilization of graphitic carbon nitride and subsequent deposition onto a filter was achieved through a simple filtration protocol. We hope that the facile access and high modularity of **PA**-**R** will promote the wide-spread application of aromatic micelles, an emergent new class of soft molecular host compounds [14–15,30–31].

## Supporting Information

File 1 General information, experimental procedures, characterization data, and copies of spectra.
