# Supplementary material for: Facile access to pyridinium-based bent aromatic amphiphiles: nonionic surface modification of nanocarbons in water
Source: Beilstein J Org Chem. 2024 Jan 8;20:32–40. doi: 10.3762/bjoc.20.5 (PMC10790643; doi:10.3762/bjoc.20.5)
Supplement: File 1 — General information, experimental procedures, characterization data, and copies of spectra. [file Beilstein_J_Org_Chem-20-32-s001.pdf]

BEILSTEIN JOURNAL OF ORGANIC CHEMISTRY

Supporting Information

for

Facile access to pyridinium-based bent aromatic amphiphiles: nonionic surface modification of nanocarbons in water

Lorenzo Catti, Shinji Aoyama and Michito Yoshizawa

Beilstein J. Org. Chem. **2024,** *20,* 32–40. doi:10.3762/bjoc.20.5

General information, experimental procedures, characterization data, and copies of spectra

License and Terms: This is a supporting information file under the terms of the Creative Commons Attribution License (https://creativecommons.org/licenses/by/4.0). Please note that the reuse, redistribution and reproduction in particular requires that the author(s) and source are credited and that individual graphics may be subject to special legal provisions.
The license is subject to the *Beilstein Journal of Organic Chemistry* terms and conditions: (https://www.beilstein-journals.org/bjoc/terms)

Table of contents

- Materials and methods, References
- Synthesis of **prePA** (^{1}H & ^{13}C NMR, ^{1}H,^{1}H COSY, and HSQC spectra)
- Synthesis of **PA-CH$_3$**, **PA-OCH$_3$**, **PA-OH**, and **PA-Im** (^{1}H & ^{13}C NMR, ^{1}H,^{1}H COSY, HSQC, and MS spectra)
- Formation of aromatic micelles (**PA-R**)$_n$ (^{1}H & ^{13}C NMR, DOSY, DLS, and UV–vis spectra)
- Formation of (**PA-R**)$_n$•(**C$_{60}$**)$_m$ (UV–vis spectra)
- Formation of (**PA-OCH$_3$**)$_n$•(***s*/*m*-CNT**)$_m$ and (**PA-OCH$_3$**)$_n$•(**GN**)$_m$
- Zeta-potential measurements of (**PA-R**)$_n$ and (**PA-OCH$_3$**)$_n$•(***s*-CNT**)$_m$
- Zeta-potential measurements of (**PA-OCH$_3$** or **PA-Im**)$_n$•(**C$_{60}$**)$_m$ under neutral/acidic conditions (UV–vis spectra)
- Water-solubilization and deposition of **g-C$_3$N$_4$** (FTIR spectra, AFM images, and photographs)
- Insolubility of nonionic **AA’** (^{1}H NMR spectrum)
- Formation of (**PA-CH$_3$**)$_n$•(**DCM**)$_m$ (UV–vis spectra)
- Interactions of (**PA-Im**)$_n$ with $PdCl_2(CH_3CN)_2$ (^{1}H NMR spectra)

Materials and methods

NMR: Bruker AVANCE-400 (400 MHz) and ASCEND-500 (500 MHz), MALDI-TOF MS: Bruker ultrafleXtreme, ESI-TOF MS: Bruker micrOTOF II, UV–vis: JASCO V-670DS, DLS: Wyatt Technology DynaPro NanoStar, AFM: Asylum Research Cypher S, FTIR: SHIMADZU IRSpirit-T, Zeta-potential: Malvern Zetasizer μV, Molecular mechanics (MM) calculation (geometry optimization): Dassault Systèmes Co., Forcite module, BIOVIA Materials Studio 2020 (version 20.1.0.5), DFT calculation: Spartan 18 Parallel Suite (Version 1.4.5, ωB97X-D; 6-31G*).

For the MM optimizations of the micelles and the host–guest composites, initial structures were constructed based on the DLS-based size analysis and the minimization of water-exposure of hydrophobic surfaces.

Solvents and reagents: TCI Co., Ltd., FUJIFILM Wako Chemical Co., Kanto Chemical Co., Inc., Sigma-Aldrich Co., and Cambridge Isotope Laboratories, Inc. Amphiphiles **AA** and **AA'** were synthesized according to ref. S1 and S5, respectively. Graphitic carbon nitride was purchased from TCI (G0539).

References

[S1] a) K. Kondo, A. Suzuki, M. Akita, M. Yoshizawa, *Angew. Chem. Int. Ed.* **2013**, *52*, 2308–2312; b) K. Kondo, A. Suzuki, M. Akita, M. Yoshizawa, *Eur. J. Org. Chem.* **2014**, *33*, 7389–7394.

[S2] N. Kishi, Z. Li, K. Yoza, M. Akita, M. Yoshizawa, *J. Am. Chem. Soc.* **2011**, *133*, 11438–11441.

[S3] K. Kondo, M. Akita, T. Nakagawa, Y. Matsuo, M. Yoshizawa, *Chem. Eur. J.* **2015**, *21*, 12741–12746.

[S4] S. Aoyama, L. Catti, M. Yoshizawa, *Angew. Chem. Int. Ed.* **2023**, *62*, e202306399.

[S5] A. Matsumoto, K. Jono, M. Akita, M. Yoshizawa, *Chem. Asian J.* **2017**, *12*, 2889–2893.

Synthesis of prePA

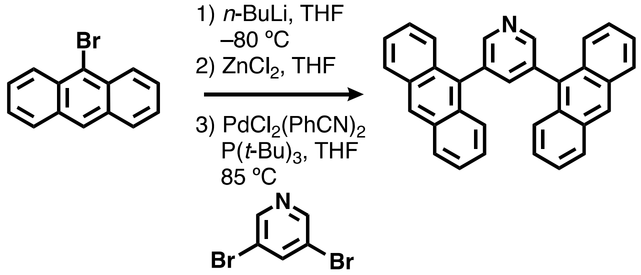

9-Bromoanthracene (3.00 g, 11.7 mmol) and dry THF (100 mL) were added to a 2-necked 300 mL glass flask filled with N_2.[S2] Then, a solution (2.7 M) of *n*-butyllithium in hexane (4.64 mL, 12.3 mmol) was added dropwise to this flask at −80 °C under N_2. After stirring the mixture at −80 °C for 40 min, a solution of $ZnCl_2$ (1.84 g, 13.5 mmol) in dry THF (10 mL) was added to the solution. The resultant mixture was further stirred at −80 °C for 2 h and then the solution was warmed to rt for 30 min to obtain 9-anthrylzinc chloride. 3,5-Dibromopyridine (1.27 g, 5.36 mmol), $PdCl_2(PhCN)_2$ (0.21 g, 0.54 mmol), and dry THF (10 mL) were added to a 2-necked 100 mL glass flask filled with N_2. A hexane solution (0.192 g/mL) of tri-*tert*-butylphosphine (1.13 mL, 1.07 mmol) was added to this flask. After stirring the mixture for 30 min at rt, the mixture was added to the 300 mL flask containing 9-anthrylzinc chloride and then the resultant solution was further stirred at 85 °C for 2 d. The mixture was concentrated under reduced pressure. Under basic conditions, the crude product was extracted with diethyl ether and CH_2Cl_2, and the combined organic phases were dried over $MgSO_4$ and concentrated under reduced pressure. The crude product was then washed with hot CH_3CN (80 °C) to afford 3,5-dianthrylpyridine (**prePA**) as a yellow solid (1.87 g, 4.33 mmol, 81%).

^{1}H NMR (400 MHz, $CDCl_3$, rt): δ 7.47-7.54 (m, 8H), 7.86 (dd, J = 8.6, 2.4 Hz, 4H), 7.92 (t, J = 2.0 Hz, 1H), 8.09 (d, J = 8.6, 2.4 Hz, 4H), 8.57 (s, 2H), 8.90 (d, J = 2.0 Hz, 2H). ^{13}C NMR (100 MHz, $CDCl_3$, rt): δ 125.3, 126.0, 126.2, 127.8, 128.7, 130.5, 131.3, 132.1, 134.4, 141.8, 150.9. FT-IR (KBr, cm^{-1}): 3026, 2979, 1623, 1519, 1443, 1401, 1350, 1160, 1014, 888, 852, 844, 788, 756. MALDI-TOF MS (dithranol): *m/z* Calcd. for $C_{33}H_{21}N$ 431.2, Found 431.1 $[M]^+$.

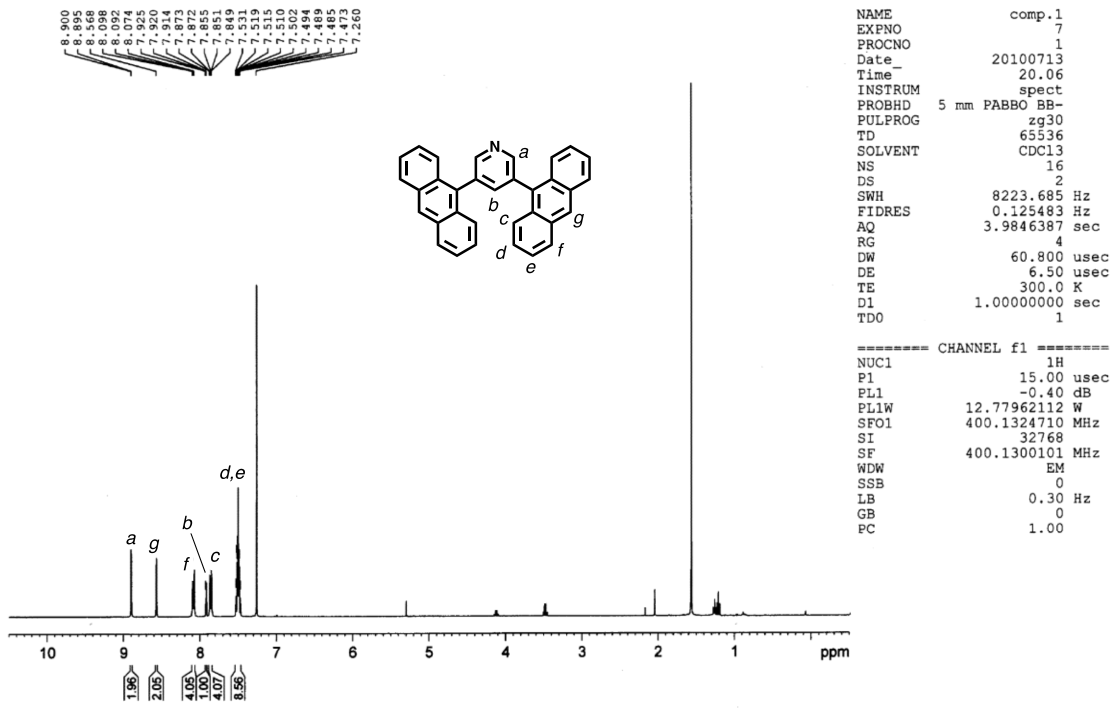

Figure S1. ^{1}H NMR spectrum (400 MHz, $CDCl_3$, rt) of **prePA**.

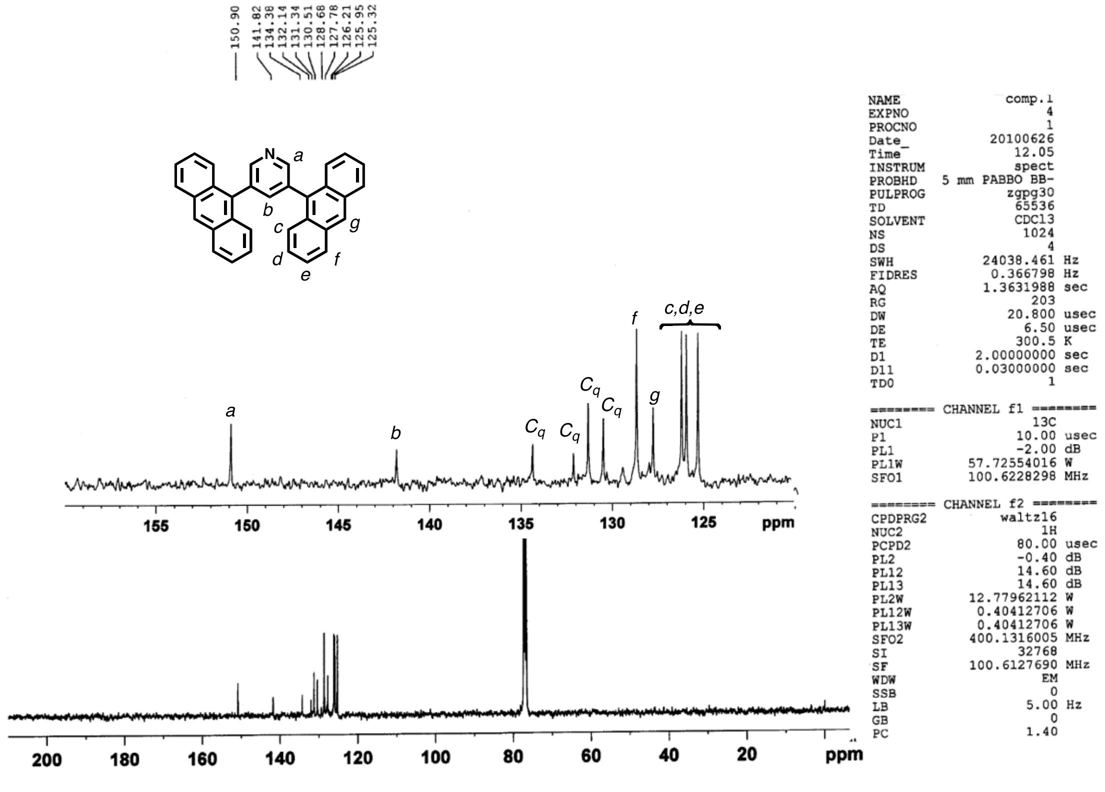

Figure S2. ^{13}C NMR spectrum (100 MHz, $CDCl_3$, rt) of **prePA**.

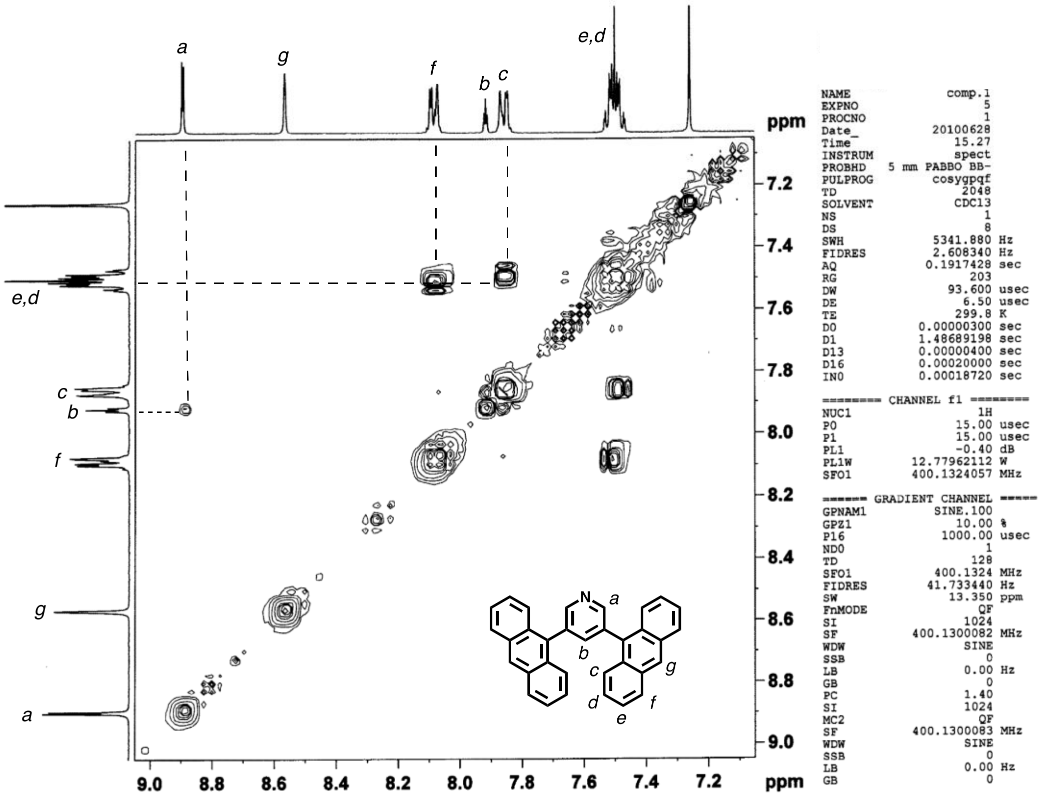

Figure S3. ^{1}H-^{1}H COSY spectrum (400 MHz, $CDCl_3$, rt) of **prePA**.

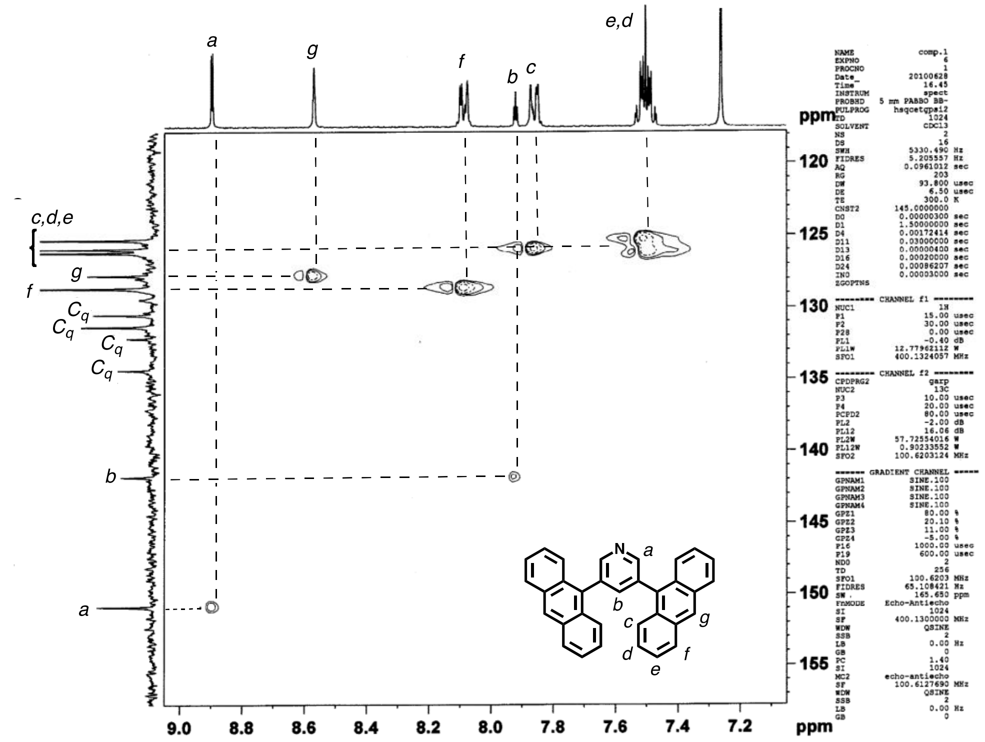

Figure S4. HSQC spectrum (400 MHz, $CDCl_3$, rt) of **prePA**.

Synthesis of PA-CH_3

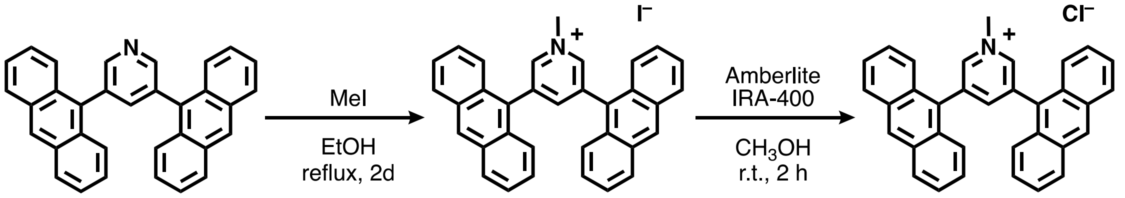

prePA (196 mg, 0.455 mmol) and EtOH (30 mL) were added to a 2-necked 200 mL glass flask. A solution of methyl iodide (2.25 mL, 36.1 mmol) in EtOH (10 mL) was added and the solution was stirred at 85 °C for 2 d. The mixture was concentrated under reduced pressure, and the resulting solid residue was washed with hexane to yield **PA-CH_3'** as a brown solid (152 mg, 0.264 mmol, 75%). **PA-CH_3'** (192 mg, 0.334 mmol), amberlite IRA-400 (2.7 g), and CH_3OH (30 mL) were added to a 100 mL glass flask and the mixture was stirred at rt for 2 h. The resultant suspension was filtered and concentrated under vacuum. The residue was washed with a mixture of CH_2Cl_2 and hexane to yield **PA-CH_3** as a yellow solid (157 mg, 0.327 mmol, 98%).

PA-CH_3': ^{1}H NMR (400 MHz, $CDCl_3$, rt): δ 4.93 (s, 3H), 7.56-7.57 (m, 4H), 7.65-7.69 (m, 4H), 7.92 (d, J = 8.8 Hz, 4H), 8.11 (d, J = 8.4 Hz, 4H), 8.61 (s, 1H), 8.67 (s, 2H), 8.90 (s, 2H). MALDI-TOF MS (dithranol): *m/z* Calcd. for $C_{33}H_{21}N$ 446.2, Found 446.2 $[M]^+$.

PA-CH_3: ^{1}H NMR (500 MHz, CD_3OD, rt): δ 4.68 (s, 3H), 7.56-7.68 (m, 8H), 7.91 (d, J = 8.4 Hz, 4H), 8.21 (d, J = 8.4 Hz, 4H), 8.76 (s, 1H), 8.80 (s, 2H), 9.36 (d, J = 1.5 Hz, 2H). ^{13}C NMR (125 MHz, CD_3OD, rt): δ 49.6, 125.9, 126.8, 128.4, 128.6, 130.1, 130.9, 131.7, 132.7, 141.3, 147.7, 151.9. FT-IR (KBr, cm^{-1}): 3355, 3047, 1630, 1496, 1445, 1270, 1018, 894, 847, 790, 739, 692. ESI-TOF MS (CH_3OH): *m/z* Calcd. for $C_{34}H_{24}N$ 446.19, Found 446.26 $[M - Cl^-]^+$.

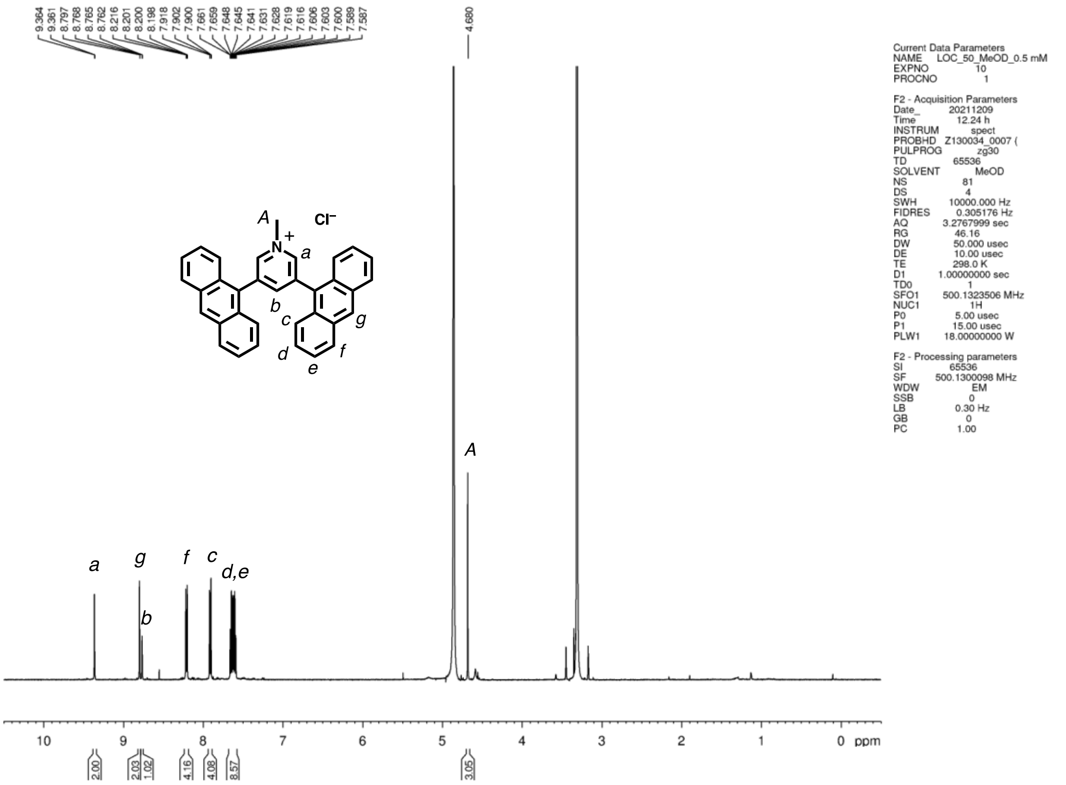

Figure S5. ^{1}H NMR spectrum (400 MHz, CD_3OD, rt) of **PA-CH$_3$**.

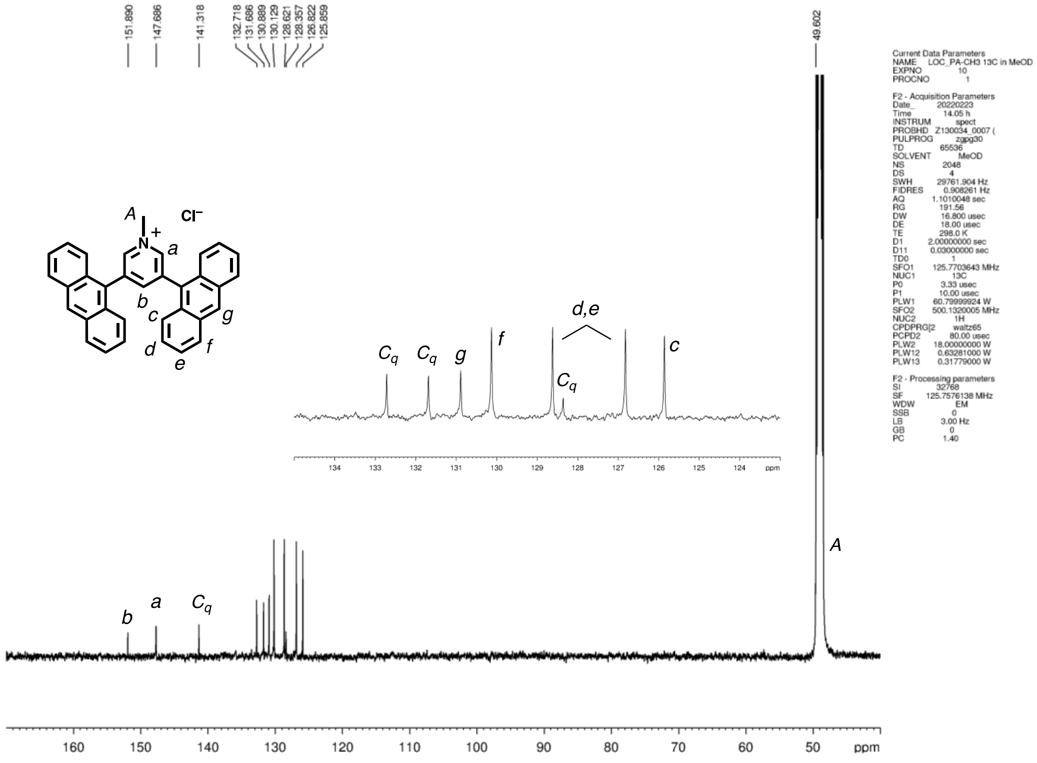

Figure S6. ^{13}C NMR spectrum (125 MHz, CD_3OD, rt) of **PA-CH$_3$**.

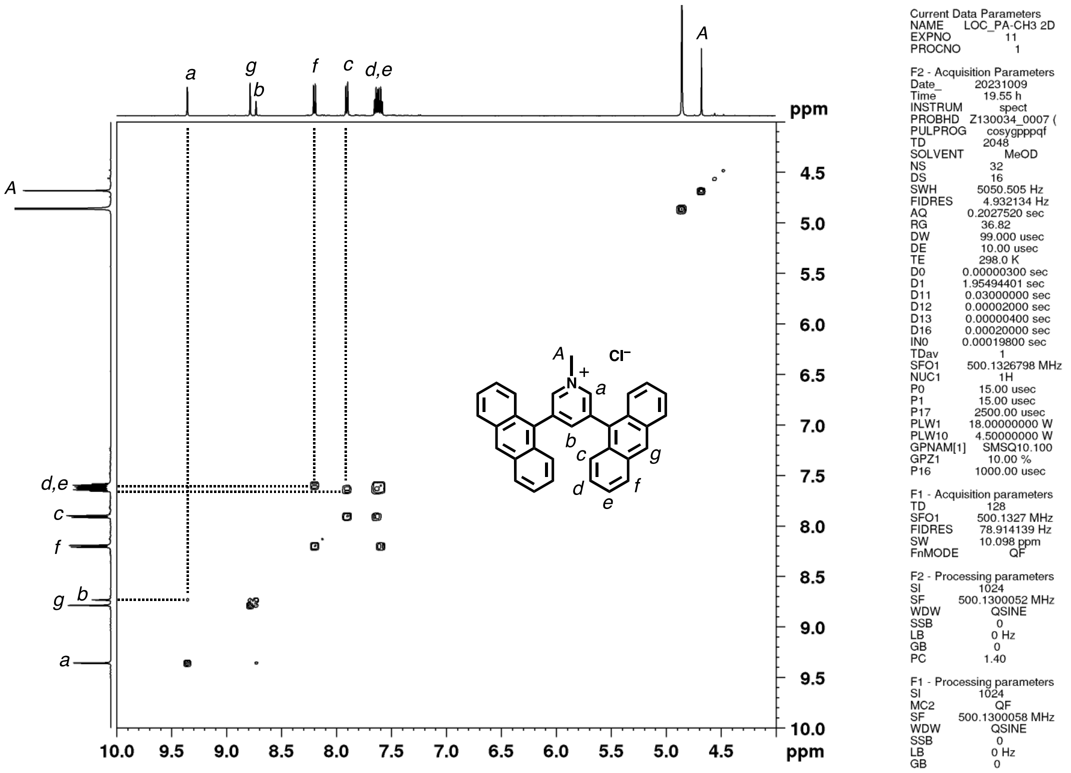

Figure S7. ^{1}H-^{1}H COSY spectrum (500 MHz, CD_3OD, rt) of **PA-CH$_3$**.

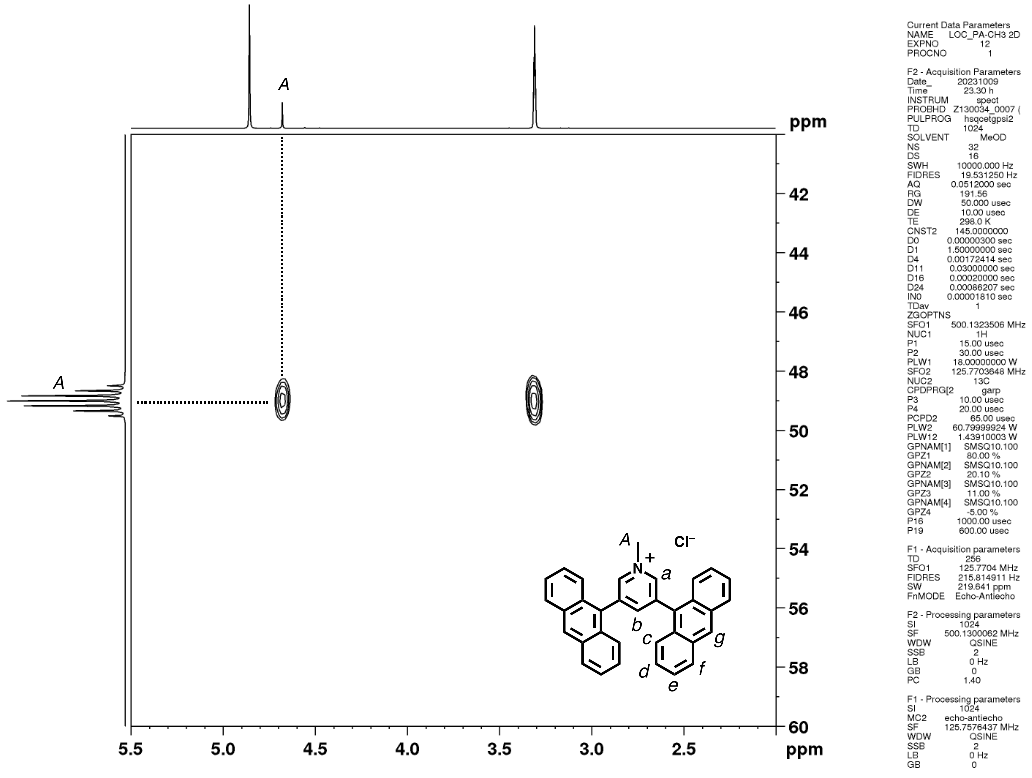

Figure S8. HSQC spectrum (500 MHz, CD_3OD, rt) of **PA-CH$_3$**.

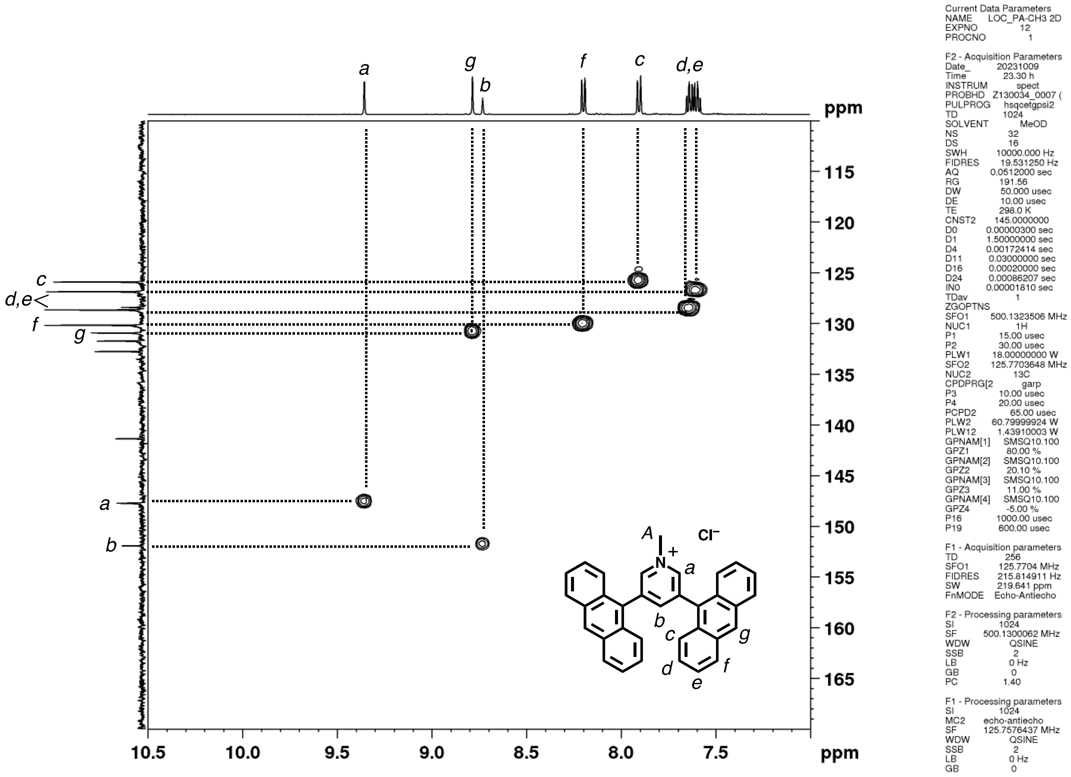

Figure S9. HSQC spectrum **(**500 MHz, CD_3OD, rt) of **PA-CH$_3$**.

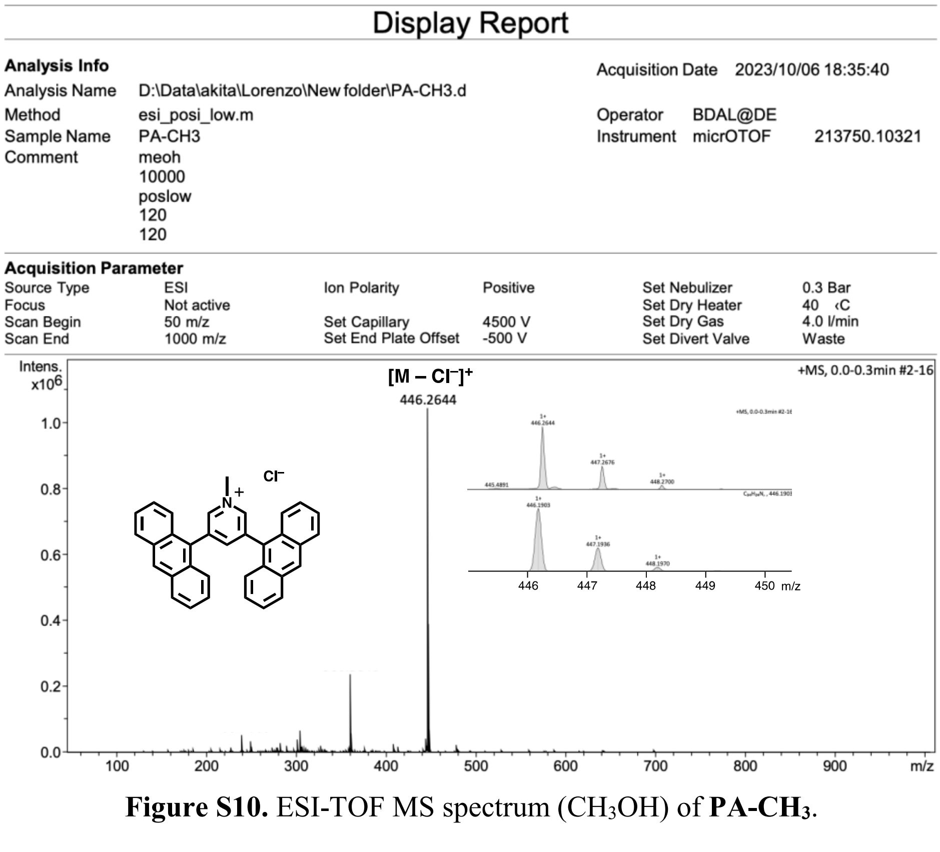

Figure S10. ESI-TOF MS spectrum (CH_3OH) of **PA-CH$_3$**.

Synthesis of PA-OCH3

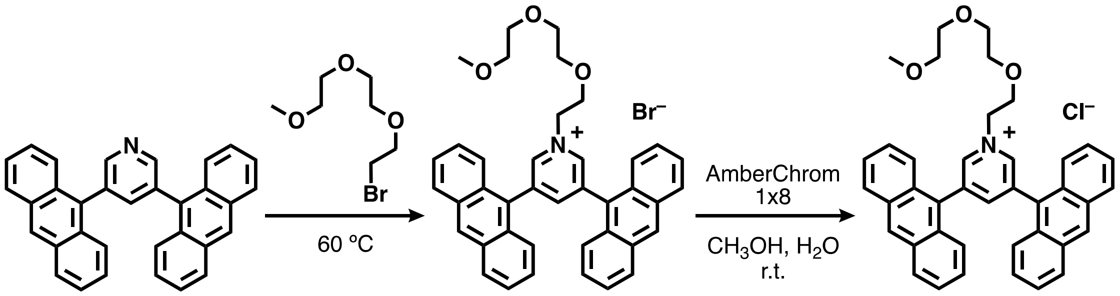

prePA (204 mg, 0.473 mmol) and 1-(2-bromoethoxy)-2-(2-methoxyethoxy)-ethane (2.50 mL, 10.1 mmol) were added to a 2-necked 10 mL glass flask filled with N_2. The resulting suspension was stirred at 60 °C for 45 h. Due to incomplete conversion, another portion of 1-(2-bromoethoxy)-2-(2-methoxyethoxy)ethane (2.00 mL, 8.08 mmol) was added and the resultant suspension was stirred at 100 °C for 3 d. Et_2O (50 mL) was added at rt and the precipitated solid was isolated by centrifugation. The crude product was washed with Et_2O to yield **PA-OCH3'** as a yellow solid (215 mg, 0.326 mmol, 69%). **PA-OCH3'** (200 mg, 0.304 mmol), AmberChrom 1x8 Cl-form (1.5 g), CH_3OH (28 mL), and H_2O (68 mL) were added to a 100 mL glass flask and the mixture was stirred at rt for 29 h. The resultant suspension was filtered and washed with H_2O, and the filtrate was concentrated under vacuum. The residue was re-dissolved in H_2O (30 mL), passed through a membrane filter (200 nm pore size) and then lyophilized to give **PA-OCH3** as a yellow solid (180 mg, 0.293 mmol, 96%).

PA-OCH3': ^{1}H NMR (400 MHz, DMSO-d_6, rt): δ 2.99 (s, 3H), 3.05 (t, J = 5.0 Hz, 2H), 3.25 (t, J = 5.0 Hz, 2H), 3.49 (t, J = 5.0 Hz, 2H), 3.70 (t, J = 5.0 Hz, 2H), 4.13 (t, J = 4.8 Hz, 2H), 4.99 (t, J = 4.8 Hz, 2H), 7.60-7.71 (m, 8H), 7.92 (d, J = 9.0 Hz, 4H), 8.27 (m, 4H), 8.91 (s, 2H), 8.96 (s, 1H), 9.54 (s, 2H).

PA-OCH3: ^{1}H NMR (500 MHz, CD_3OD, rt): δ 2.89 (s, 3H), 2.89-2.91 (m, 2H), 3.11-3.15 (m, 2H), 3.53-3.56 (m, 2H), 3.78-3.82 (m, 2H), 4.18 (t, J = 4.9 Hz, 2H), 5.06 (t, J = 4.9 Hz, 2H), 7.57-7.67 (m, 8H), 7.87 (d, J = 8.9 Hz, 4H), 8.20 (d, J = 8.5 Hz, 4H), 8.75 (t, J = 1.6 Hz, 1H), 8.77 (s, 2H), 9.44 (d, J = 1.6 Hz, 2H). ^{13}C NMR (125 MHz, CD_3OD, rt): δ 58.7, 62.9, 70.3, 71.0, 71.4, 71.7, 72.5, 125.8, 126.9, 128.4, 128.7, 130.2, 131.0, 131.7, 132.7, 141.0, 147.5, 152.4. FT-IR (ATR, cm^{-1}): 3056, 2876, 1622, 1447, 1294, 1187, 1096, 897, 848, 791, 741, 703. ESI-TOF MS (CH_3OH): *m/z* Calcd. for $C_{40}H_{36}NO_3$ 578.27, Found 578.24 $[M - Cl^-]^+$.

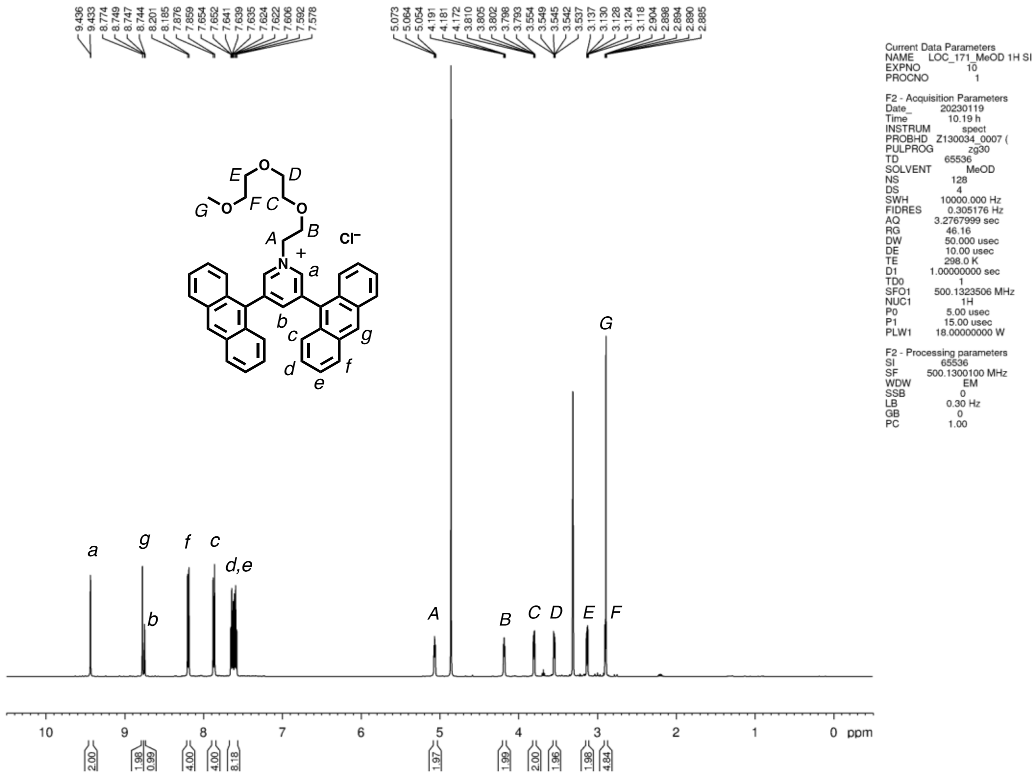

Figure S11. ^{1}H NMR spectrum (500 MHz, CD_3OD, rt) of **PA-OCH$_3$**.

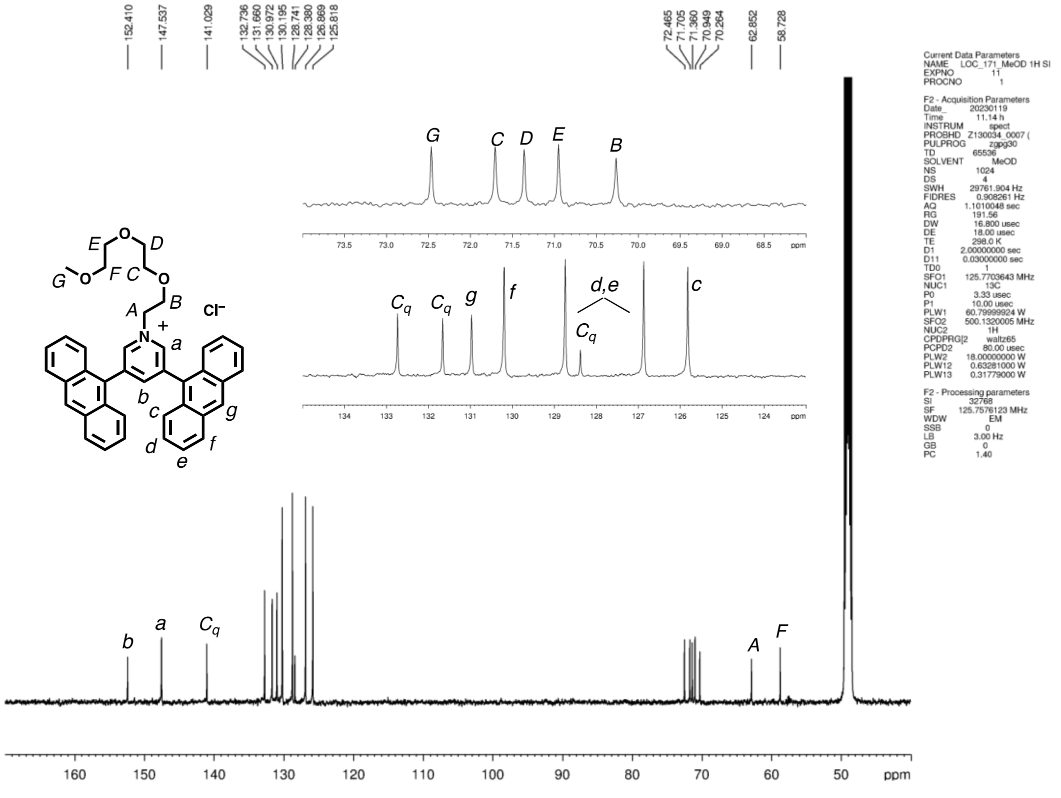

Figure S12. ^{13}C NMR spectrum (125 MHz, CD_3OD, rt) of **PA-OCH$_3$**.

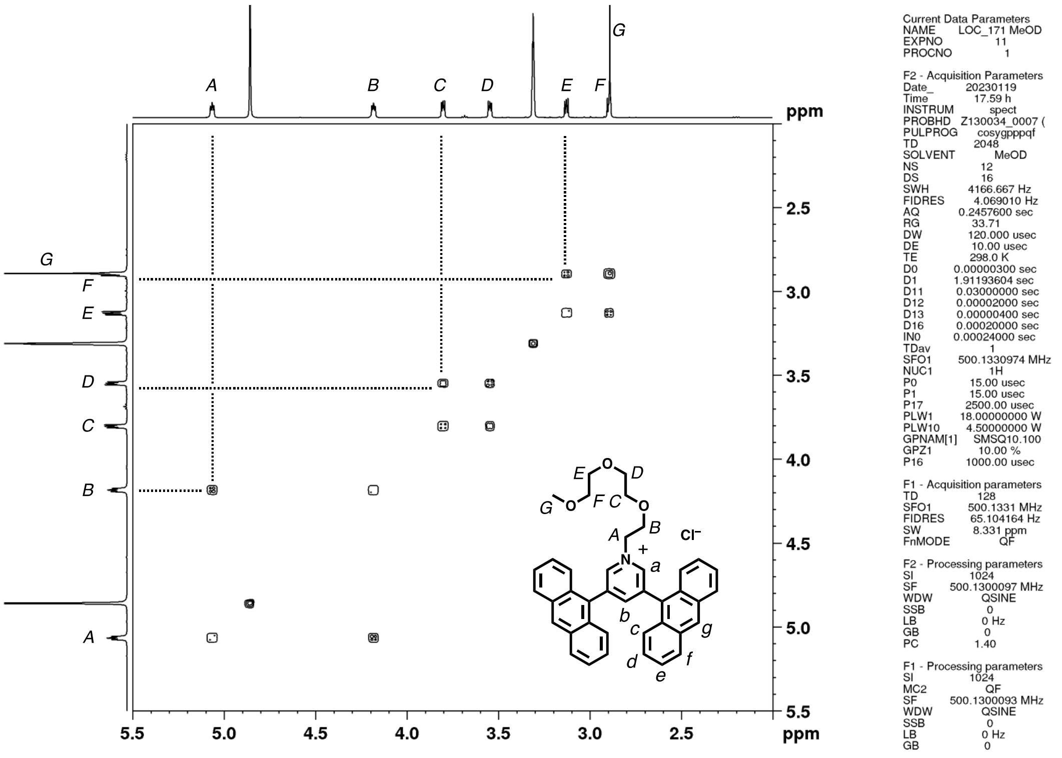

Figure S13. ^{1}H-^{1}H COSY spectrum **(**500 MHz, CD_3OD, rt) of **PA-OCH$_3$**.

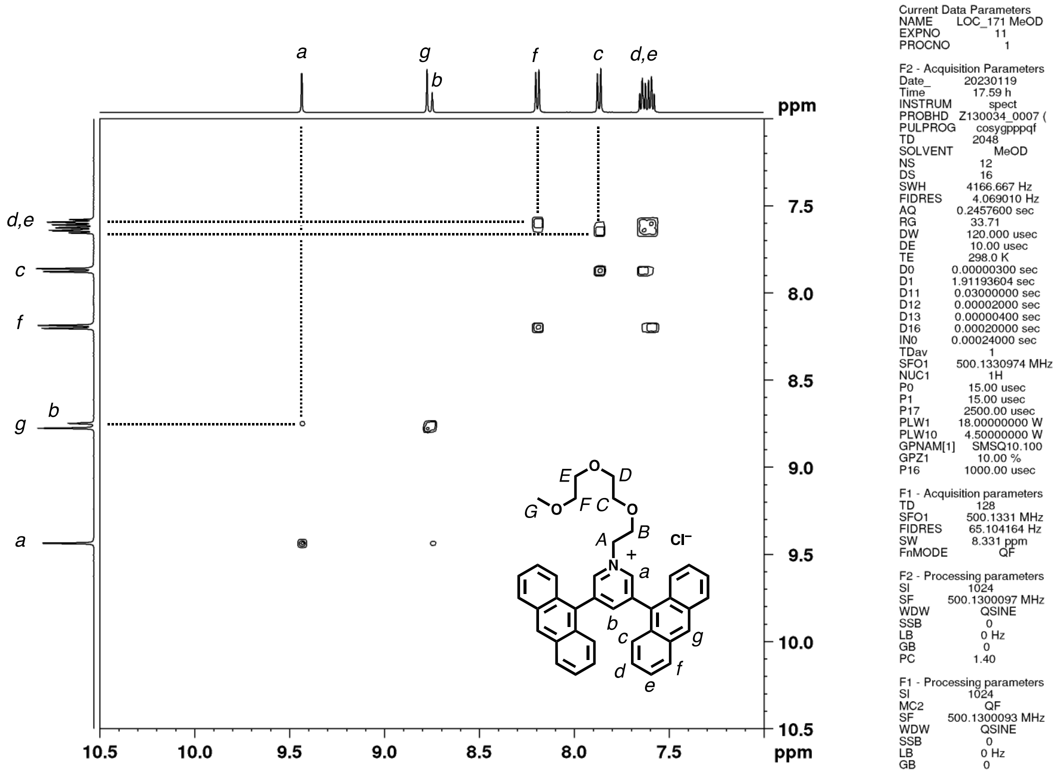

Figure S14. ^{1}H-^{1}H COSY spectrum **(**500 MHz, CD_3OD, rt) of **PA-OCH$_3$**.

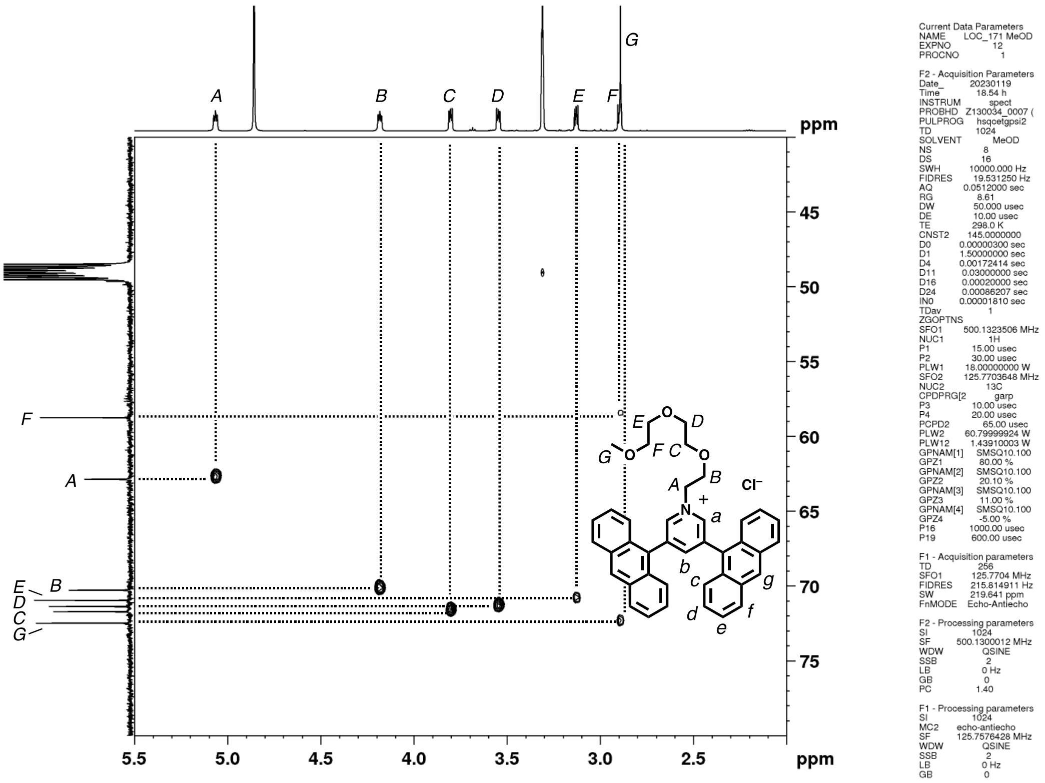

Figure S15. HSQC spectrum (500 MHz, CD_3OD, rt) of **PA-OCH$_3$**.

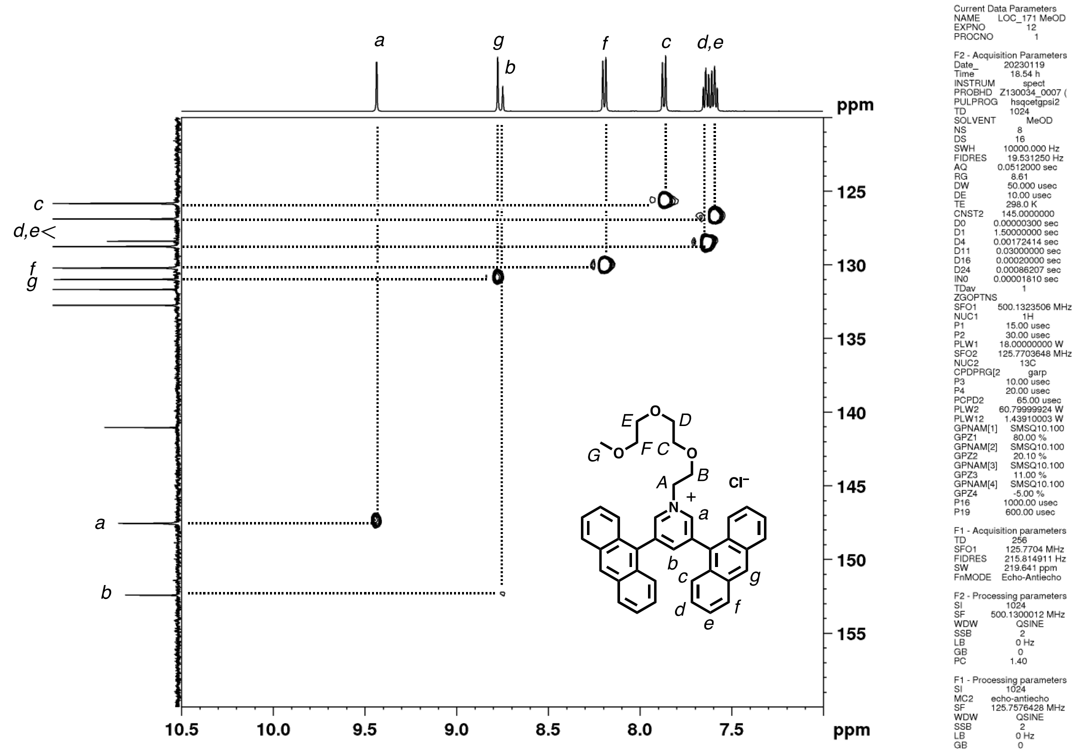

Figure S16. HSQC spectrum (500 MHz, CD_3OD, rt) of **PA-OCH$_3$**.

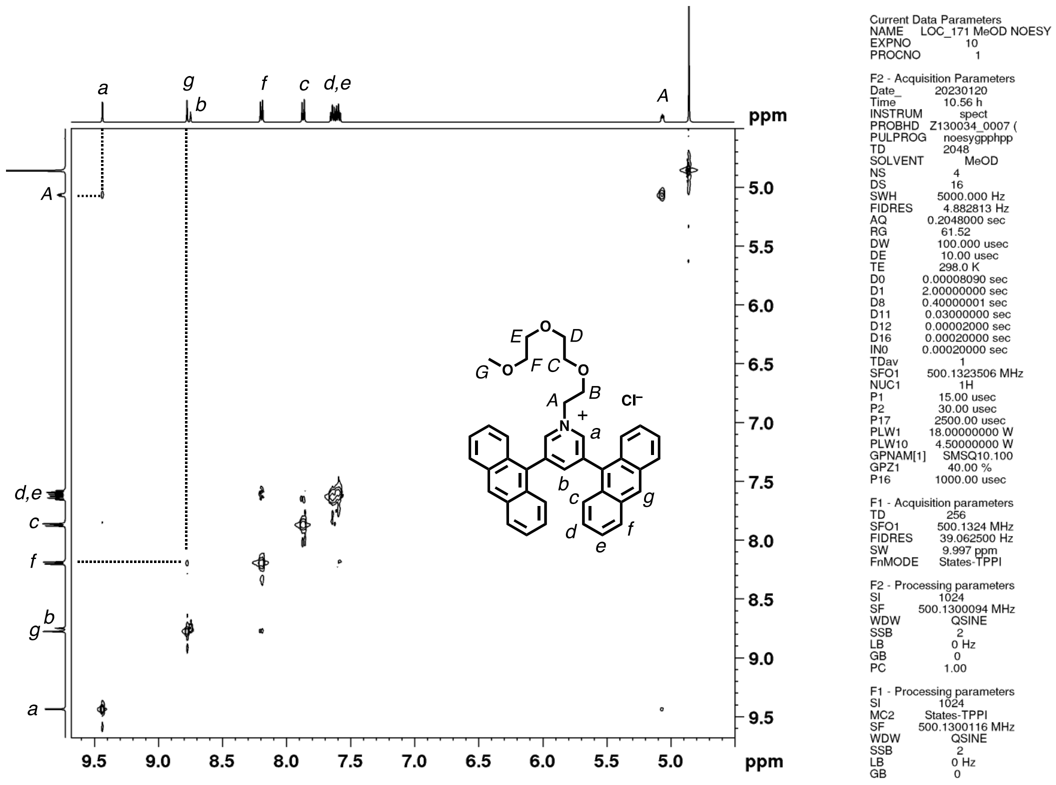

Figure S17. NOESY spectrum (500 MHz, CD_3OD, rt) of **PA-OCH$_3$**.

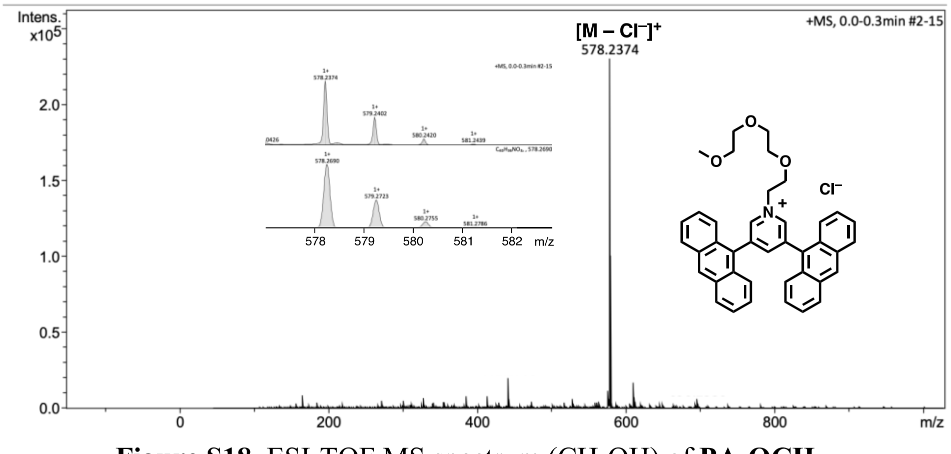

Display Report

Analysis Info

Analysis Name D:\Data\akita\Lorenzo\LOC_171\LOC_171_W1MeOD000001.d
Method Pd Kusaba01.m
Sample Name LOC_171_W1 MeOD
Comment MeOD
10000 times NMR diluted
200
100
60 deg

Acquisition Date 2022/10/20 15:35:37
Operator BDAL@DE
Instrument micrOTOF 213750.10321

Acquisition Parameter

Source Type	ESI	Ion Polarity	Positive	Set Nebulizer	3.0 Bar
Focus	Not active			Set Dry Heater	30 ‹C
Scan Begin	50 m/z	Set Capillary	4500 V	Set Dry Gas	6.0 l/min
Scan End	3000 m/z	Set End Plate Offset	-500 V	Set Divert Valve	Waste

Figure S18. ESI-TOF MS spectrum (CH_3OH) of **PA-OCH$_3$**.

Synthesis of PA-OH

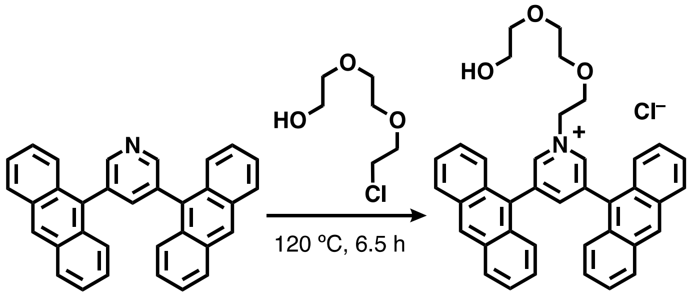

prePA (204 mg, 0.473 mmol) and 2-[2-(2-chloroethoxy)ethoxy]ethanol (12.0 mL, 82.6 mmol) were added to a 2-necked 30 mL glass flask filled with N_2. The mixture was stirred at 120 °C for 6.5 h. To the clear orange solution was added Et_2O (400 mL) and the resulting suspension was stored at rt overnight. The precipitate was isolated via filtration and then reprecipitated from CH_3OH/Et_2O (3 times) to yield **PA-OH** as a yellow solid (120 mg, 0.200 mmol, 42%).

PA-OH: ^{1}H NMR (500 MHz, CD_3OD, rt): δ 3.13-3.16 (m, 2H), 3.16-3.19 (m, 2H), 3.55-3.60 (m, 2H), 3.78-3.84 (m, 2H), 4.20 (t, J = 4.9 Hz, 2H), 5.08 (t, J = 4.9 Hz, 2H), 7.56-7.69 (m, 8H), 7.87 (d, J = 8.8 Hz, 4H), 8.21 (d, J = 8.8 Hz, 4H), 8.79 (s, 2H), 8.81 (s, 1H), 9.45 (d, J = 1.4, 2H). ^{13}C NMR (125 MHz, CD_3OD, rt): δ 61.8, 62.8, 70.3, 71.4, 71.7, 73.4, 125.8, 126.9, 128.3, 128.8, 130.2, 131.0, 131.7, 132.7, 141.1, 147.5, 152.5. FT-IR (ATR, cm^{-1}): 3254, 3040, 2927, 2867, 1687, 1445, 1113, 1071, 1036, 894, 789, 740, 699. ESI-TOF MS (CH_3OH): *m/z* Calcd. for $C_{39}H_{34}NO_3$ 564.25, Found 564.35 $[M - Cl^-]^+$.

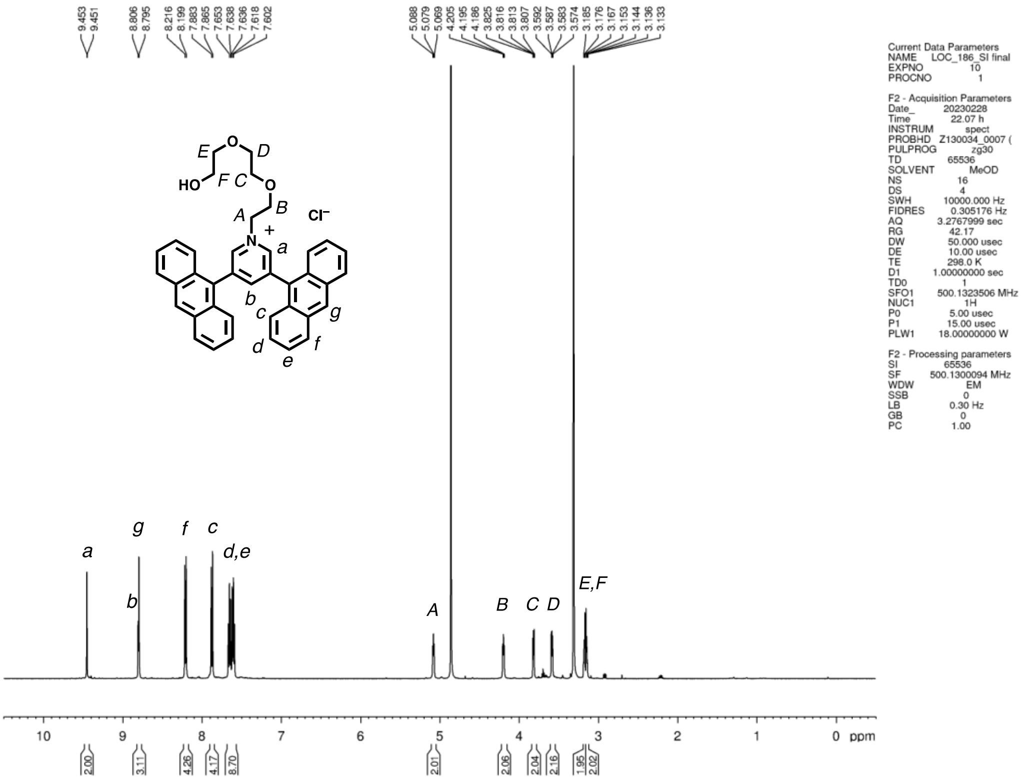

Figure S19. ^{1}H NMR spectrum (500 MHz, CD_3OD, rt) of **PA-OH**.

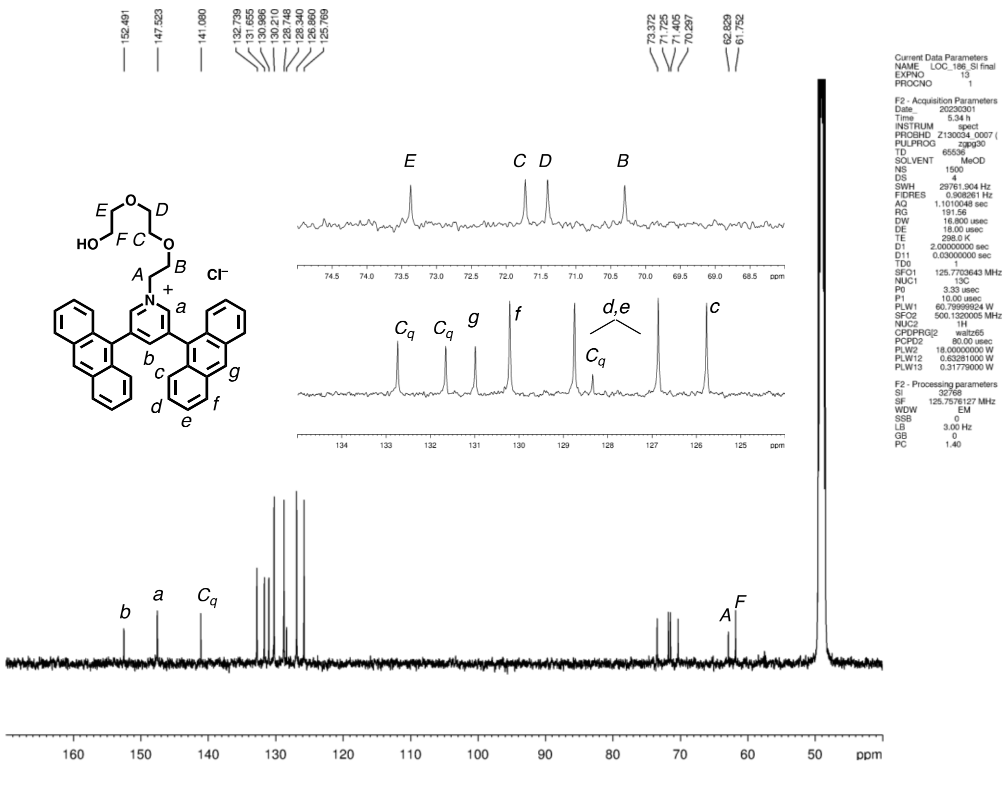

Figure S20. ^{13}C NMR spectrum (125 MHz, CD_3OD, rt) of **PA-OH**.

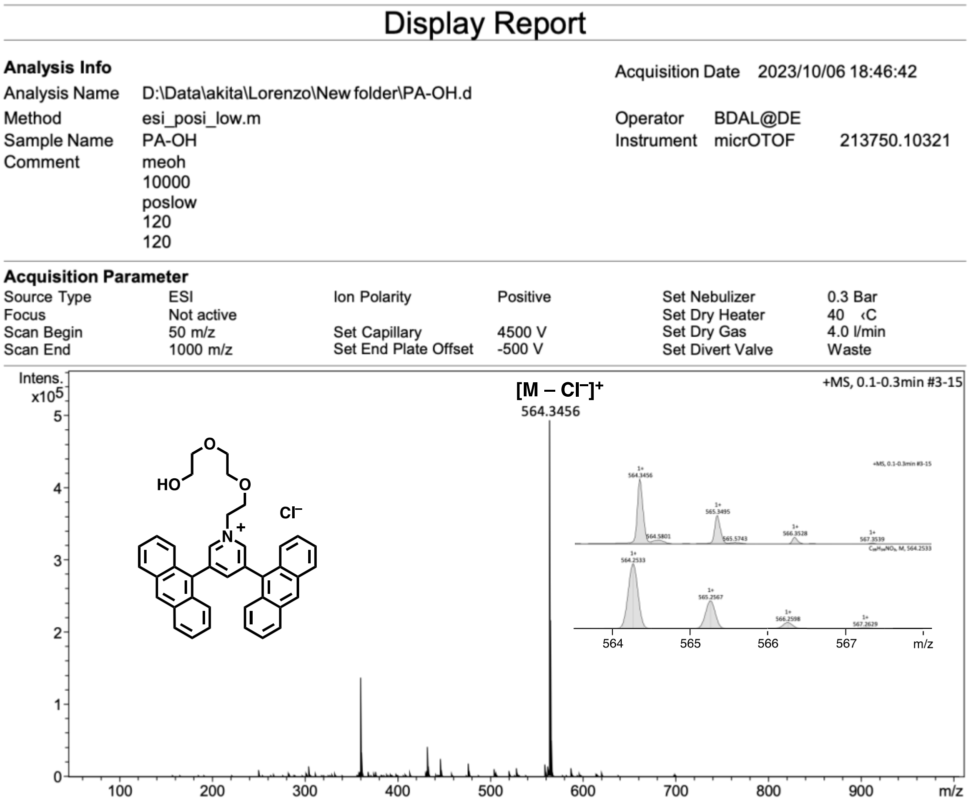

Figure S21. ESI-TOF MS spectrum (CH_3OH) of **PA-OH**.

Synthesis of PA-Im

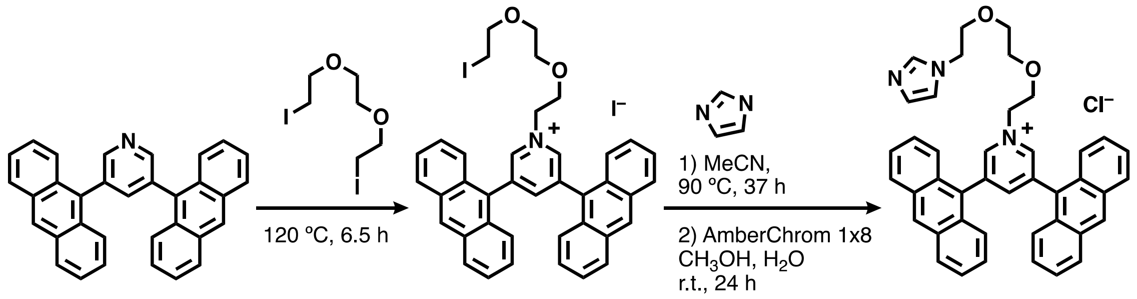

prePA (297 mg, 0.688 mmol) and 1,2-bis(2-iodoethoxy)ethane (5.30 mL, 24.2 mmol) were added to a 2-necked 20 mL glass flask filled with N_2. The mixture was stirred at 80 °C for 53 h. To the clear brown solution was added Et_2O (45 mL) and the precipitated solid was isolated by centrifugation. The crude product was washed with Et_2O (4 times) to yield **PA-I** as a yellow solid (482 mg, 0.601 mmol, 87%). **PA-I** (200 mg, 0.250 mmol), imidazole (230 mg, 3.38 mmol), and dry MeCN (34 mL) were added to a 2-necked 100 mL glass flask filled with N_2. The mixture was then stirred at 90 °C for 37 , followed by concentrated of the mixture under reduced pressure. The crude product was re-precipitated from MeCN/Et_2O (4 times). After the residue was redissolved in H_2O and

extracted with CH_2Cl_2 (3 times), the resultant solution was dried over $MgSO_4$, filtered, and concentrated to yield **PA-Im'** as a yellow solid (132 mg, 0.178 mmol). **PA-Im'** (132 mg, 0.178 mmol), AmberChrom 1x8 Cl-form (1.0 g), CH_3OH (10 mL), and H_2O (30 mL) were added to a 100 mL glass flask and the mixture was stirred at rt for 24 h. The resultant suspension was vacuum-filtered and washed with H_2O and the obtained filtrate was concentrated under vacuum. After the residue was re-dissolved in H_2O (15 mL), the solution was passed through a membrane filter (200 nm pore size) and then lyophilized to give **PA-Im** as a yellow solid (77.4 mg, 0.119 mmol, 48% over 2 steps).

PA-I: ^{1}H NMR (400 MHz, CD_3CN, rt): δ 2.69 (t, J = 7.3 Hz, 2H), 3.22 (t, J = 6.6 Hz, 2H), 3.45-3.54 (m, 2H), 3.67-3.76 (m, 2H), 4.15 (t, J = 5.3 Hz, 2H), 4.92 (t, J = 5.3 Hz, 2H), 7.59-7.69 (m, 8H), 7.88 (d, J = 8.9 Hz, 4H), 8.22 (d, J = 8.0 Hz, 4H), 8.74 (s, 1H), 8.82 (s, 2H), 9.12 (d, J = 1.6 Hz, 2H).

PA-Im': ^{1}H NMR (400 MHz, CD_3CN, rt): δ 3.25-3.31 (m, 2H), 3.43-3.47 (m, 2H), 3.61 (t, J = 5.9 Hz, 2H), 3.64-3.69 (m, 2H), 4.06 (t, J = 5.1 Hz, 2H), 6.59 (s, 1H), 6.66 (s, 1H), 7.25 (s, 1H), 7.57-7.68 (m, 8H), 7.821-7.91 (m, 2H), 8.17-8.27 (m, 2H), 8.73 (s, 1H), 8.82 (s, 2H), 9.07 (s, 2H). ESI-TOF MS (CH_3OH): *m/z* Calcd. for $C_{39}H_{33}INO_2$ 674.16, Found 674.22 $[M - I^-]^+$.

PA-Im: ^{1}H NMR (500 MHz, CD_3OD, rt): δ 3.36 (t, J = 5.1 Hz, 2H), 3.53-3.58 (m, 2H), 3.72-3.77 (m, 4H), 4.12 (t, J = 4.9 Hz, 2H), 5.04 (t, J = 4.9 Hz, 2H), 6.64 (s, 1H), 6.78 (s, 1H), 7.57 (s, 1H), 7.57-7.67 (m, 8H), 7.86 (d, J = 8.5 Hz, 4H), 8.21 (d, J = 8.0 Hz, 4H), 8.80 (s, 2H), 8.83 (s, 1H), 9.34 (d, J = 1.4 Hz, 2H). ^{13}C NMR (125 MHz, CD_3OD, rt): δ 48.0, 62.9, 70.2, 70.8, 71.5, 71.5, 121.0, 125.7, 126.9, 127.3, 128.3, 128.8, 130.2, 131.0, 131.6, 132.7, 138.4, 141.1, 147.4, 152.6. FT-IR (ATR, cm^{-1}): 3360, 3046, 2879, 1621, 1445, 1106, 1076, 893, 789, 736, 670. ESI-TOF MS (CH_3OH): *m/z* Calcd. for $C_{42}H_{36}N_3O_2$ 614.28, Found 614.38 $[M - Cl^-]^+$.

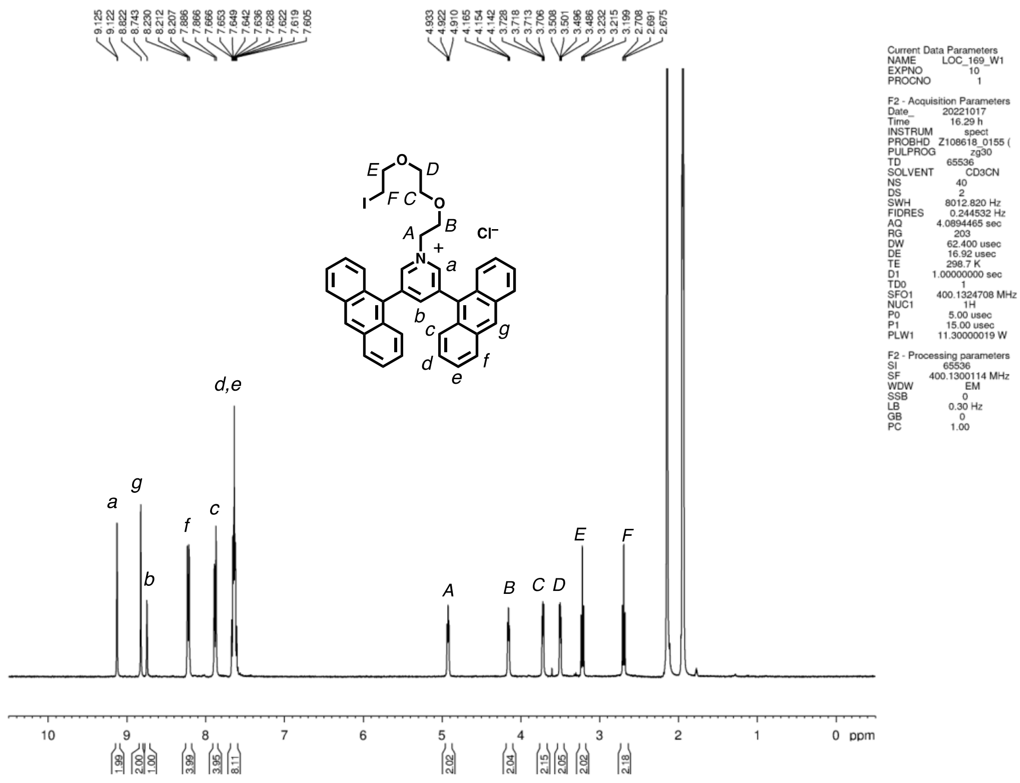

Figure S22. ^{1}H NMR spectrum (400 MHz, CD_3CN, rt) of **PA-I**.

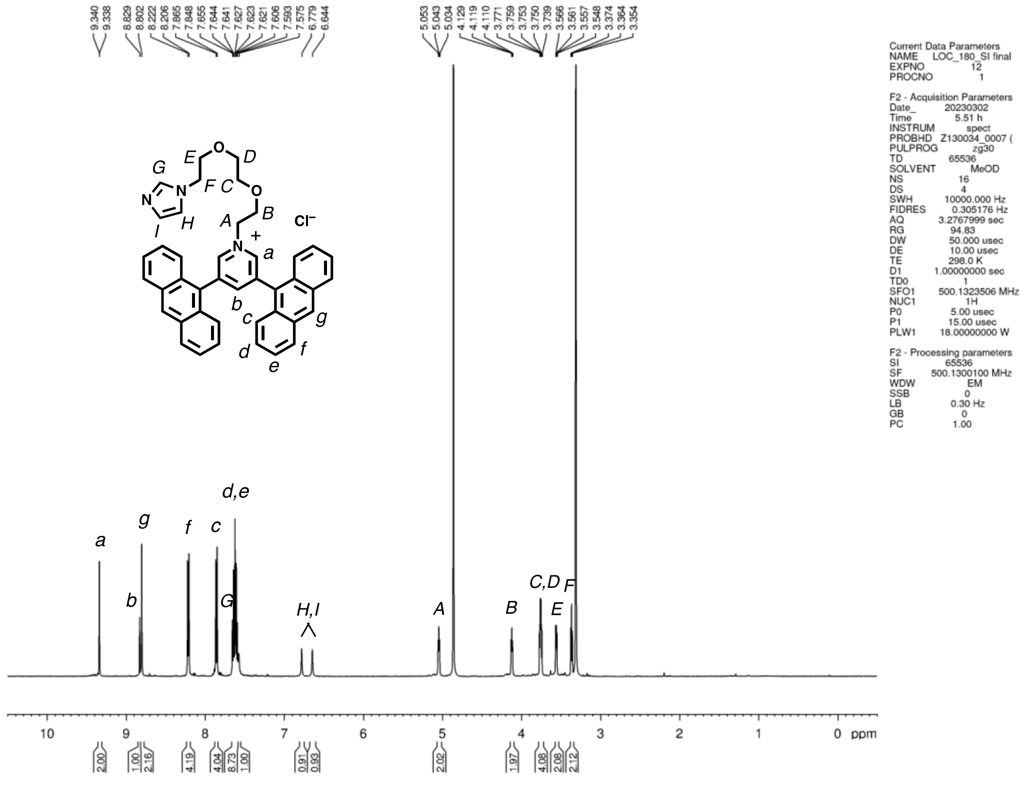

Figure S23. ^{1}H NMR spectrum (400 MHz, CD_3OD, rt) of **PA-Im**.

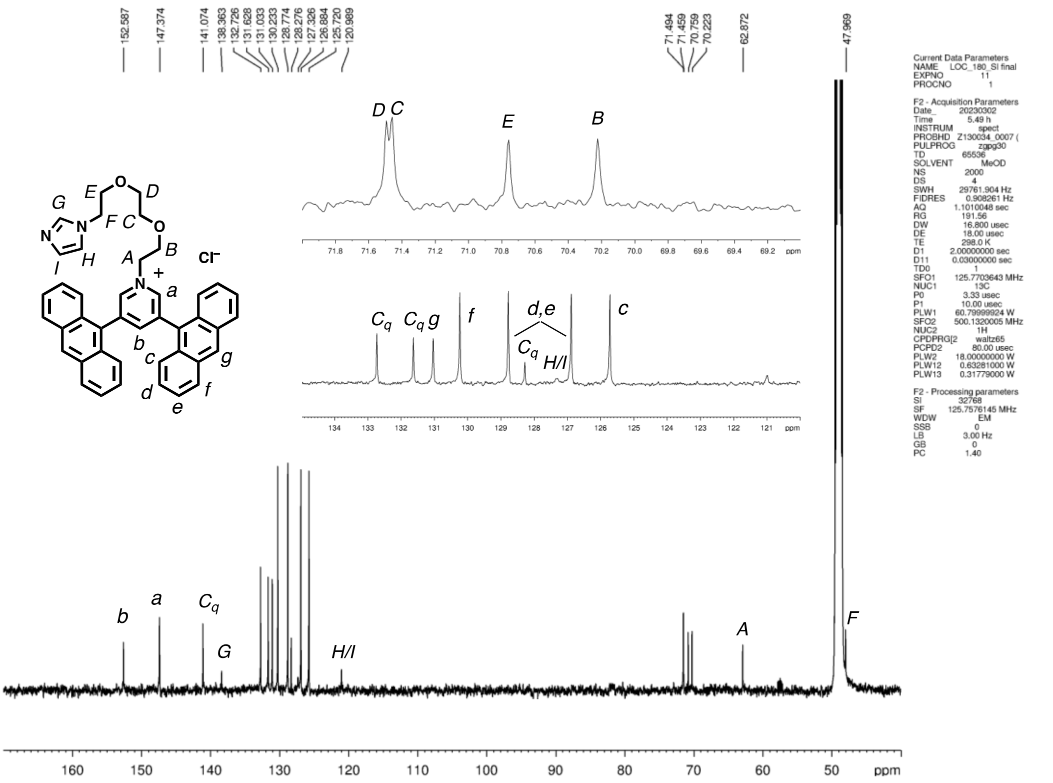

Figure S24. ^{13}C NMR spectrum (125 MHz, CD_3OD, rt) of **PA-Im**.

Display Report

Analysis Info

Analysis Name	D:\Data\akita\Lorenzo\New folder\PA-Im.d	Acquisition Date	2023/10/06 18:55:14
Method	esi_posi_low.m	Operator	BDAL@DE
Sample Name	PA-Im	Instrument	micrOTOF 213750.10321
Comment	meoh<br>10000<br>poslow<br>120<br>120		

Acquisition Parameter

Source Type	ESI	Ion Polarity	Positive	Set Nebulizer	0.3 Bar
Focus	Not active			Set Dry Heater	40 ‹C
Scan Begin	50 m/z	Set Capillary	4500 V	Set Dry Gas	4.0 l/min
Scan End	1000 m/z	Set End Plate Offset	-500 V	Set Divert Valve	Waste

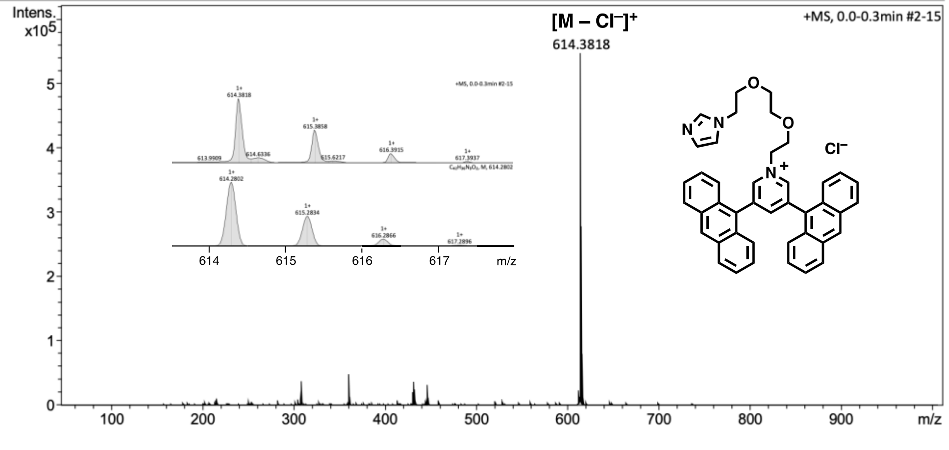

Figure S25. ESI-TOF MS spectrum (CH_3OH) of **PA-Im**.

Formation of aromatic micelles (PA-R)$_n$

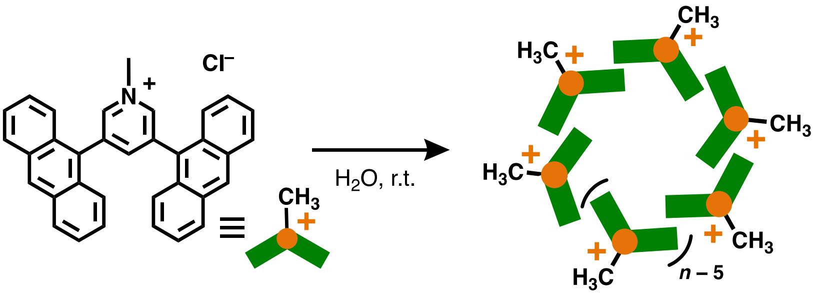

Amphiphile **PA-CH$_3$** (2.1 mg, 4.3 μmol) and water (8.6 mL) were added to a glass vial (14 mL). After short bath-sonication, the formation of (**PA-CH$_3$**)$_n$ was confirmed by ^{1}H and DOSY NMR, DLS, and UV–vis analyses. On the basis of the DOSY and DLS data, molecular modeling studies (molecular mechanics; Forcite module, Materials Studio, version 5.5.3) indicated that the structure of (**PA-CH$_3$**)$_n$ is mainly composed of six molecules of **PA-CH$_3$**. In a similar manner, aromatic micelles (**PA-OCH$_3$**)$_n$, (**PA-OH**)$_n$, and (**PA-Im**)$_n$ were prepared from amphiphiles **PA-OCH$_3$**, **PA-OH**, and (**PA-Im**)$_n$, respectively.

(**PA-CH$_3$**)$_n$: ^{1}H NMR (400 MHz, D_2O, r.t., 0.5 mM based on **PA-CH$_3$**, DMSO-d_6 as an external standard): δ 3.91 (s, 3H), 5.50 (s, 1H), 6.24-6.39 (m, 4H), 6.39-6.50 (m, 4H), 6.61-6.72 (m, 4H), 7.00-7.13 (m, 4H), 7.22-7.36 (m, 2H), 7.94-8.07 (m, 2H). DOSY NMR (500 MHz, D_2O, 25 °C, 0.9 mM based on **PA-CH$_3$**): $D = 2.30 \times 10^{-10}$ m^2 s^{-1}.

(**PA-OCH$_3$**)$_n$: ^{1}H NMR (500 MHz, D_2O, r.t., 1.0 mM based on **PA-OCH$_3$**, TMS as external standard): δ 2.44-2.51 (m, 2H), 2.71 (s, 3H), 2.81-2.88 (m, 2H), 3.32-3.36 (m, 2H), 3.61-3.68 (m, 2H), 3.96-4.02 (m, 2H), 4.85-4.82 (m, 2H), 5.97 (s, 1H), 6.79 (d, J = 8.9 Hz, 4H), 6.97 (t, 7.8 Hz, 4H), 7.20 (t, 7.2 Hz, 4H), 7.57 (d, J = 8.5 Hz, 4H), 7.75 (s, 2H), 8.57 (s, 2H). ^{13}C NMR (125 MHz, D_2O, r.t., 10.0 mM based on **PA-OCH$_3$**, TMS as external standard): δ 57.8, 61.6, 68.6, 69.3, 69.6, 70.2, 70.5, 123.4, 125.3, 125.7, 127.4, 129.0, 129.1, 129.6, 130.1, 138.5, 145.2, 149.2.

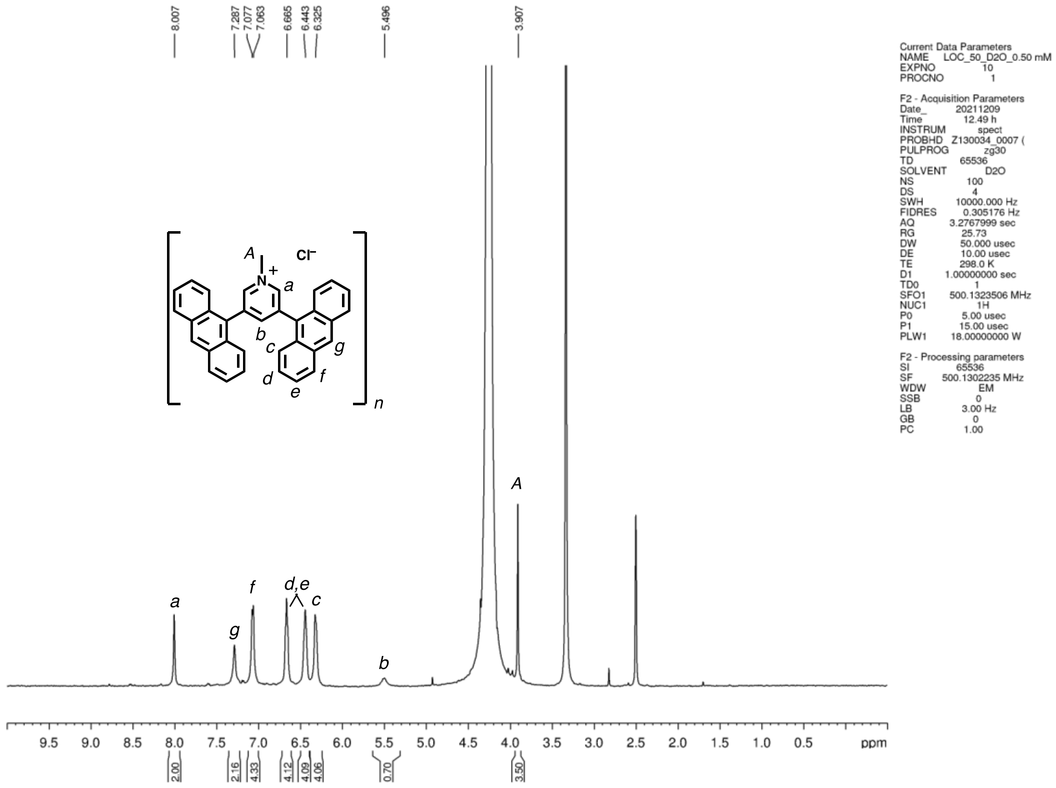

Figure S26. ^{1}H NMR spectrum (500 MHz, D_2O, rt, 0.5 mM based on **PA-CH$_3$**) of (**PA-CH$_3$**)$_n$.

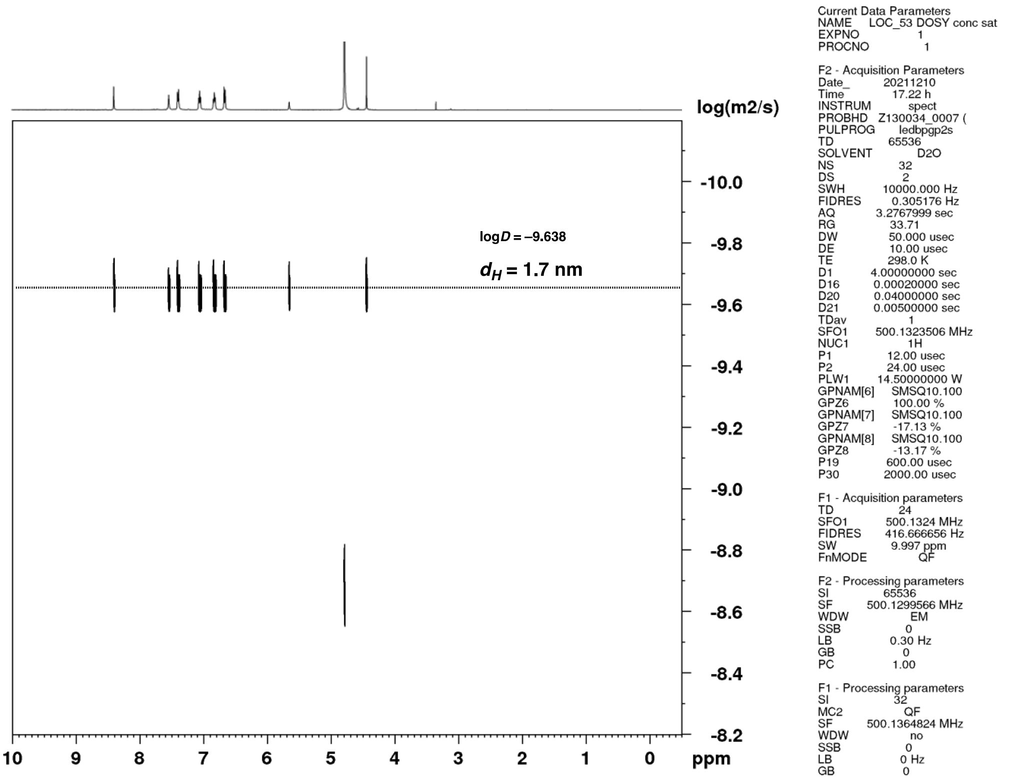

Figure S27. DOSY NMR spectrum (500 MHz, D_2O, 0.9 mM based on **PA-CH$_3$**, 25 °C) of (**PA-CH$_3$**)$_n$.

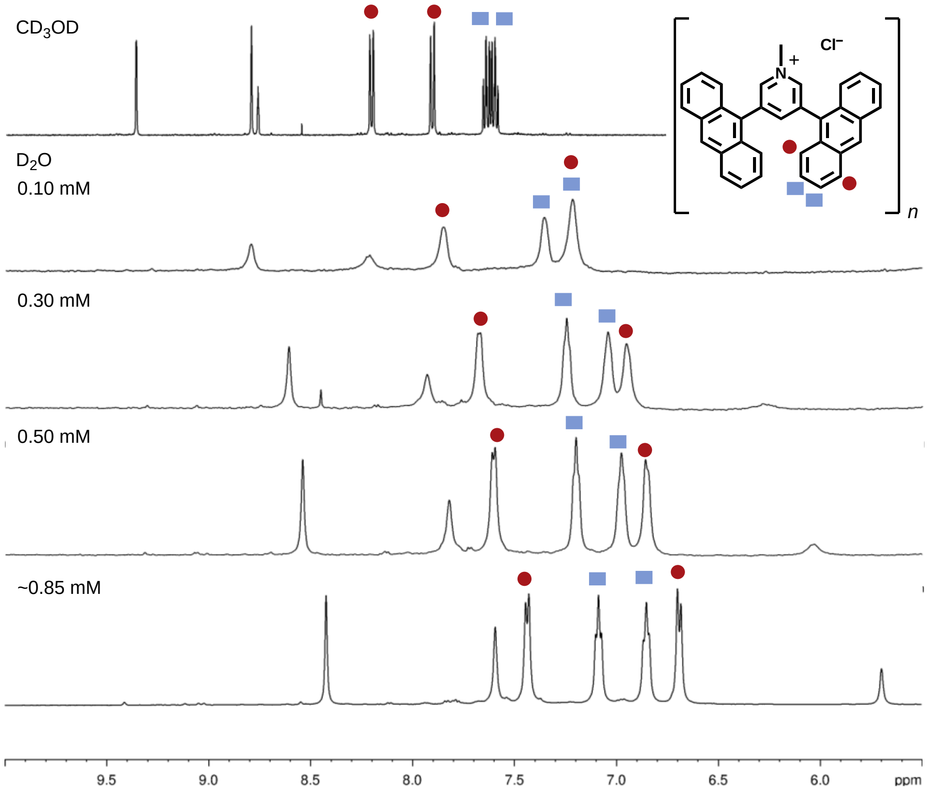

Figure S28. Concentration-dependent ^{1}H NMR spectra (500 MHz, D_2O, rt, 0.85–0.10 mM based on **PA-CH$_3$**) of (**PA-CH$_3$**)$_n$.

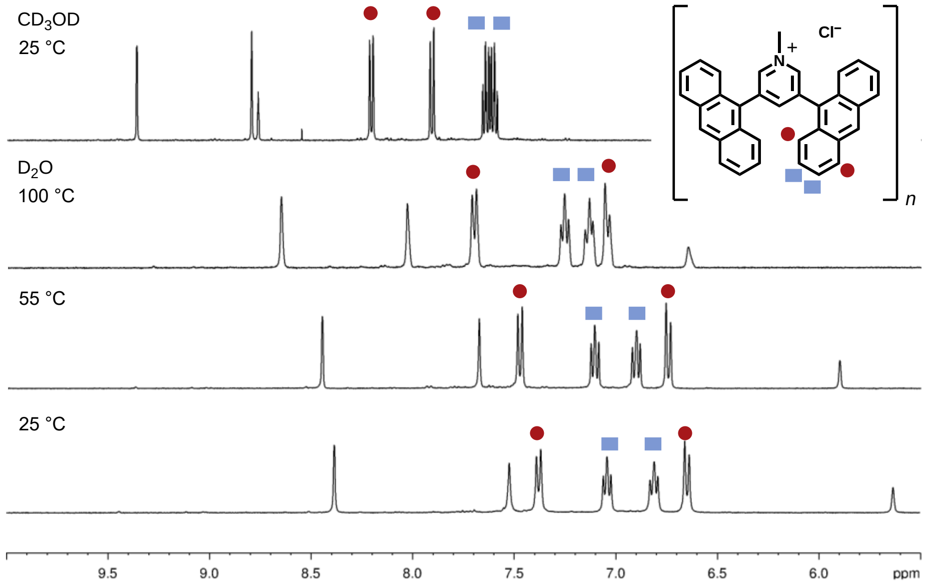

Figure S29. Temperature-dependent ^{1}H NMR spectra (500 MHz, D_2O, 0.85 mM based on **PA-CH$_3$**, DMSO (1 μL) as internal standard) of (**PA-CH$_3$**)$_n$.

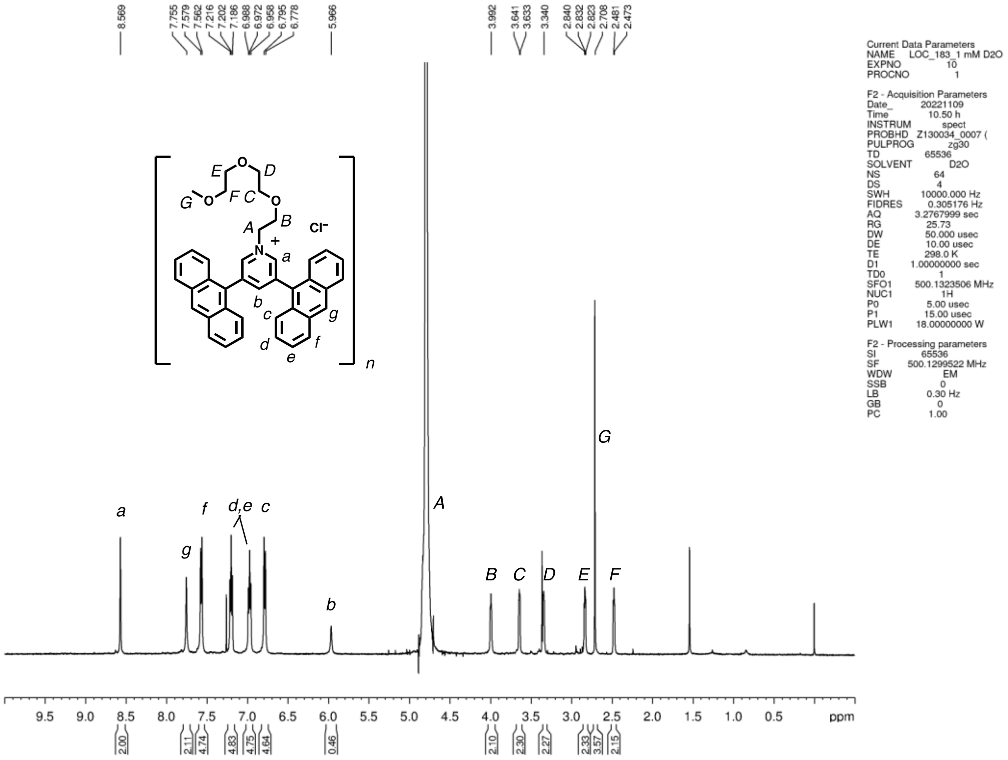

Figure S30. ^{1}H NMR spectrum (500 MHz, D_2O, rt, 1.0 mM based on **PA-OCH$_3$**, TMS as external standard) of (**PA-OCH$_3$**)$_n$.

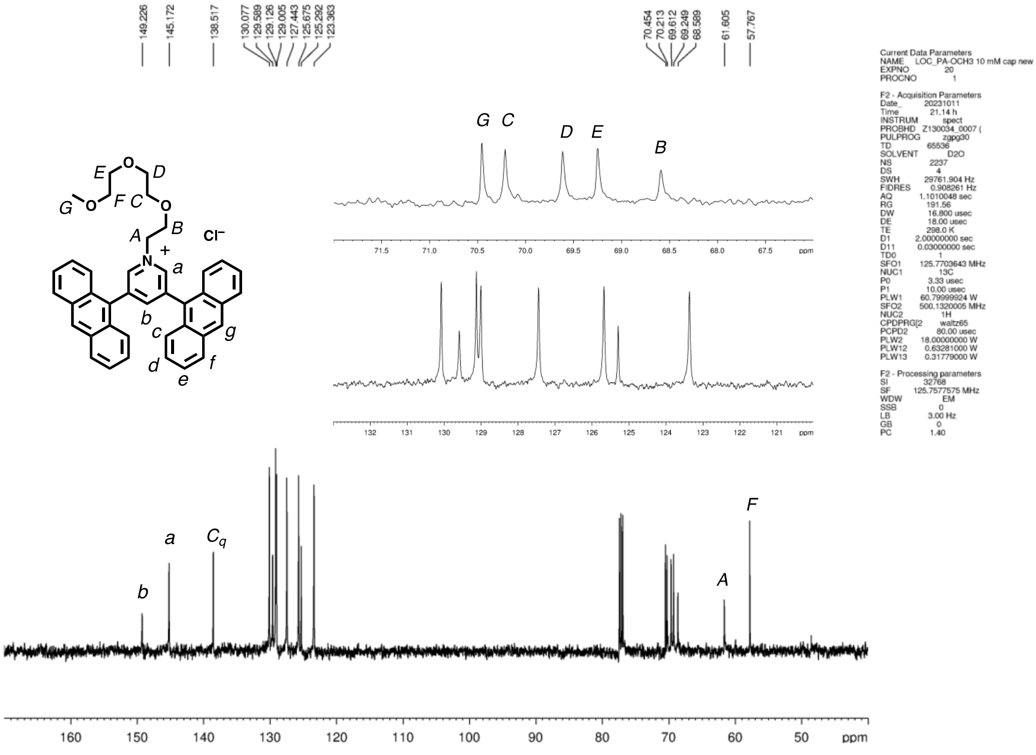

Figure S31. ^{13}C NMR spectrum (500 MHz, D_2O, rt, 10.0 mM based on **PA-OCH$_3$**, TMS as external standard) of (**PA-OCH$_3$**)$_n$.

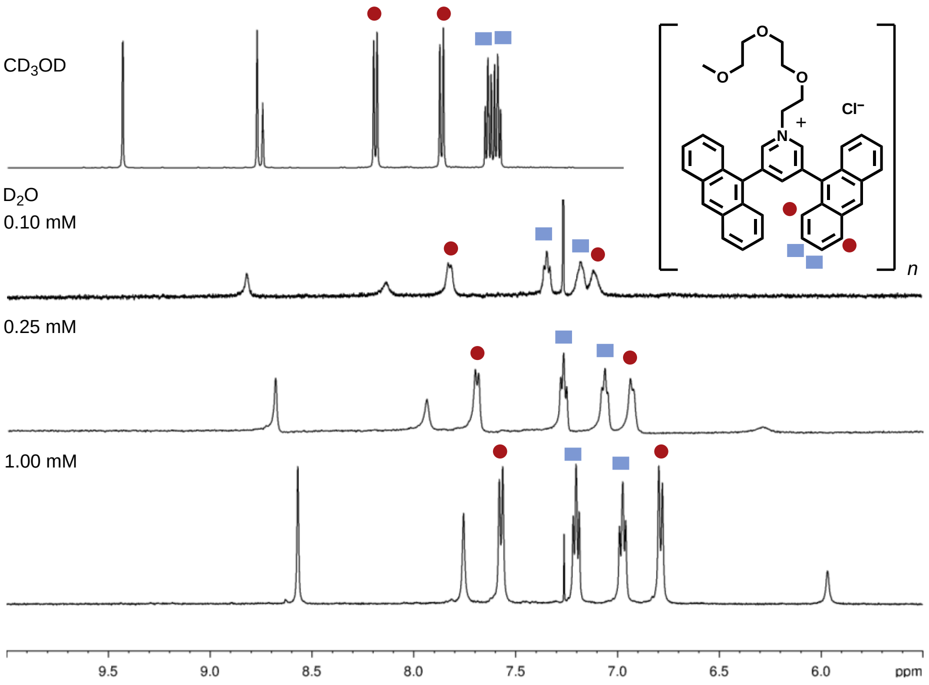

Figure S32. Concentration-dependent ^{1}H NMR spectra (500 MHz, D_2O, rt, 1.00–0.10 mM based on **PA-OCH$_3$**) of (**PA-OCH$_3$**)$_n$.

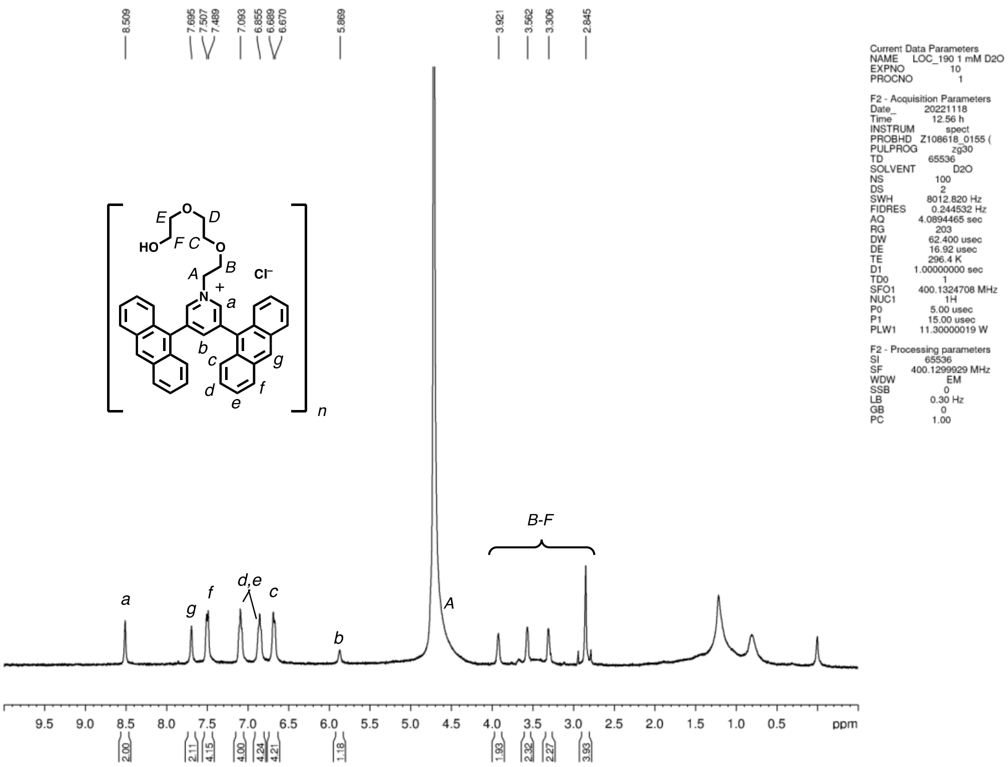

Figure S33. ^{1}H NMR spectrum (500 MHz, D_2O, rt, 1.0 mM based on **PA-OH**) of (**PA-OH**)$_n$.

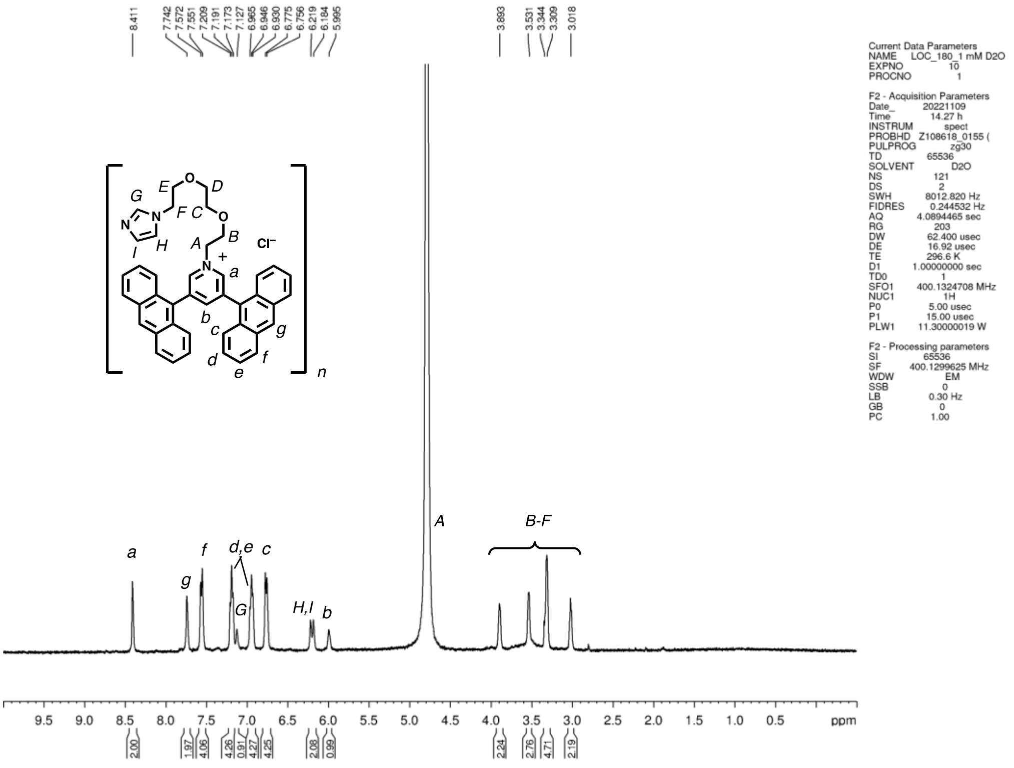

Figure S34. ^{1}H NMR spectrum (500 MHz, D_2O, rt, 1.0 mM based on **PA-Im**) of (**PA-Im**)$_n$.

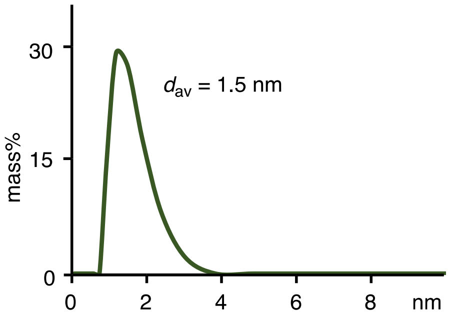

Figure S35. Particle size distribution of (**PA-OH**)$_n$ by DLS analysis (H_2O, rt, 1.0 mM based on **PA-OH**,).

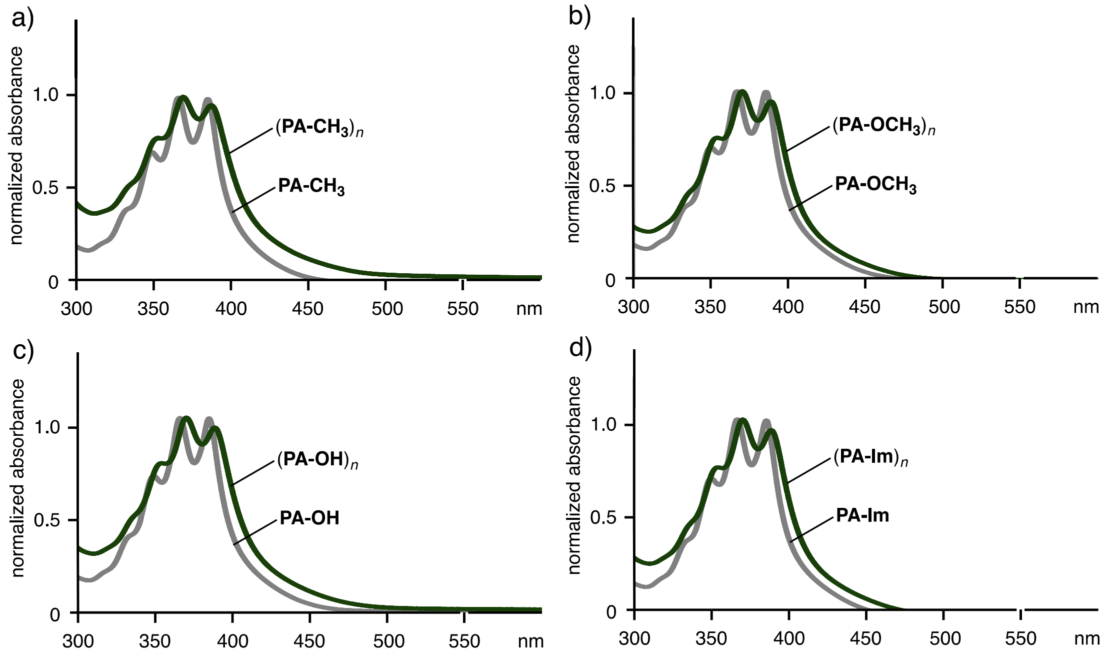

Figure S36. UV–vis spectra (rt, 0.5 mM or 1.0 mM based on **PA-R**) of a) (**PA-CH$_3$**)$_n$, b) (**PA-OCH$_3$**)$_n$, c) (**PA-OH**)$_n$, and d) (**PA-Im**)$_n$ in H_2O and the amphiphiles in CH_3OH.

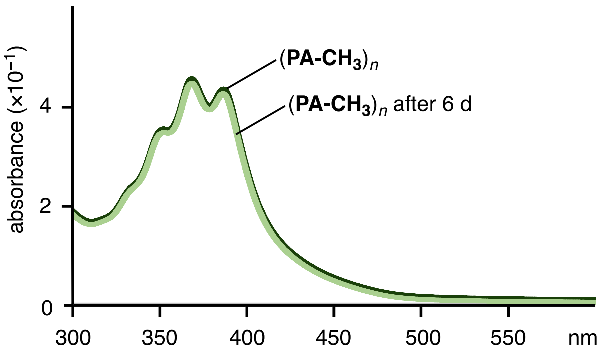

Figure S37. UV–vis spectra of (**PA-CH$_3$**)$_n$ (H_2O, rt, 0.5 mM based on **PA-CH$_3$**) directly after preparation and after storing at r.t. for 6 d in the dark.

Formation of (PA-R)$_n$•(C_{60})$_m$

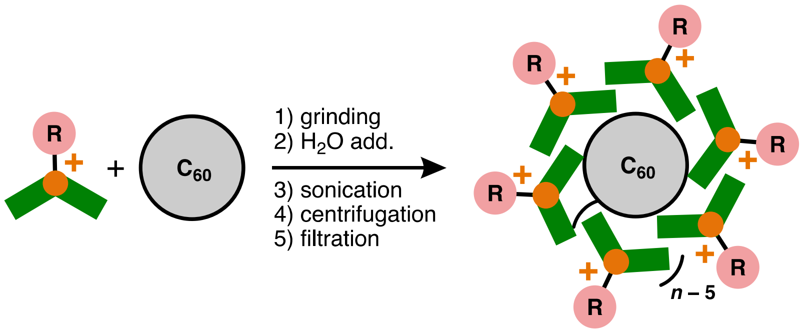

A mixture of **PA-CH3** (1.3 mg, 2.6 µmol) and **C_{60}** (1.4 mg, 1.1 µmol) was ground for 3 min by using an agate mortar and pestle.[S3] After addition of H_2O (2.6 mL), the suspension was sonicated (40 kHz, 150 W) for 10 min with a probe sonicator, centrifuged (16,000*g*) for 10 min, and then filtrated by a membrane filter (pore size: 200 nm) to give a yellow solution of (**PA-CH3**)$_n$•(**C_{60}**)$_m$. The solubilization of **C_{60}** was confirmed by UV–vis analysis. In the same way, aqueous solutions of (**PA-OCH3**)$_n$•(**C_{60}**)$_m$, (**PA-OH**)$_n$•(**C_{60}**)$_m$, (**PA-Im**)$_n$•(**C_{60}**)$_m$, (**AA**)$_n$•(**C_{60}**)$_m$, and (**SDS**)$_n$•(**C_{60}**)$_m$ were obtained by the treatment of **C_{60}** with **PA-OCH3**, **PA-OH**, **PA-Im**, **AA**, and **SDS** (sodium dodecyl sulfate), respectively. The **C_{60}** concentration (0.18 mM) of the resultant aqueous (**PA-Im**)$_n$•(**C_{60}**)$_m$ was estimated by UV–vis analysis (with a calibration curve method) in organic solvent (i.e. toluene) after the lyophilization of the isolated product and removal of **PA-Im** via washing with CH_3OH. On the basis of the DLS analysis of (**PA-Im**)$_n$•(**C_{60}**)$_m$, the optimized structure of (**PA-Im**)$_5$•**C_{60}** was obtained by the geometry optimization with molecular mechanics (MM) calculations (Forcite module, Materials Studio, version 5.5.3).

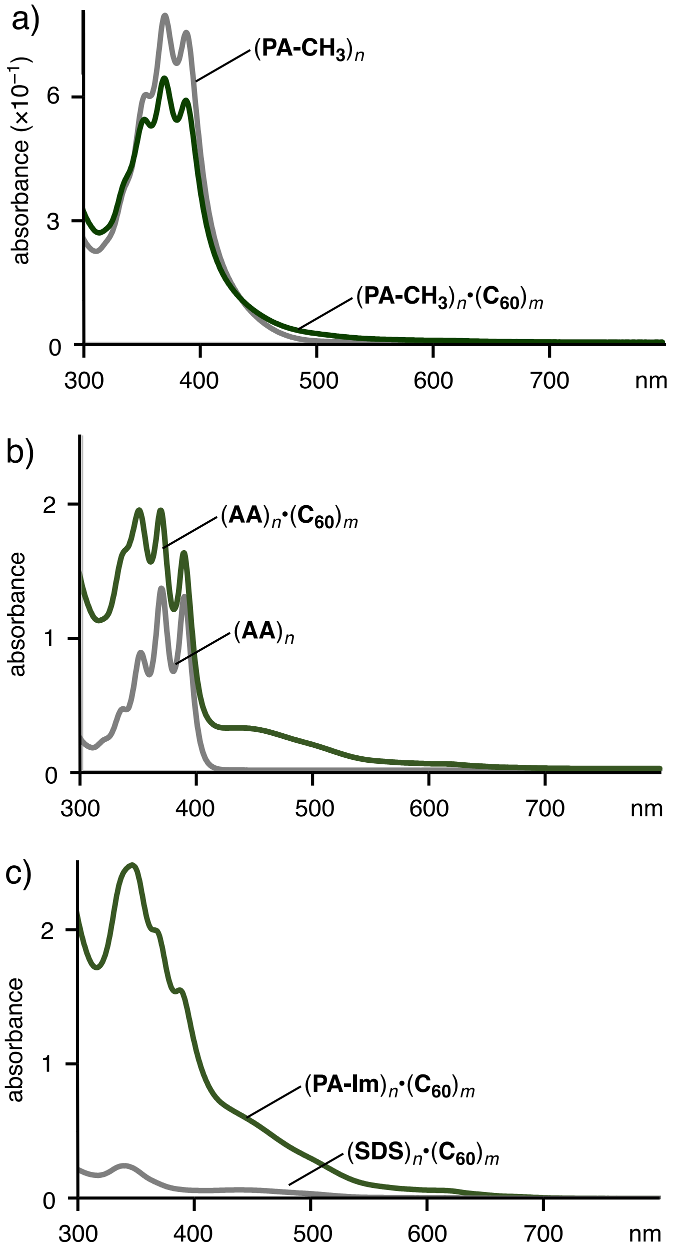

Figure S38. UV–vis spectra (H_2O, rt, 0.9 mM based on **PA-CH$_3$**, 1.0 mM based on **AA**, **PA-Im** or **SDS**) of a) (**PA-CH$_3$**)$_n$•(**C$_{60}$**)$_m$ and (**PA-CH$_3$**)$_n$, b) (**AA**)$_n$•(**C$_{60}$**)$_m$ and (**AA**)$_n$, and c) (**PA-Im**)$_n$•(**C$_{60}$**)$_m$ and (**SDS**)$_n$•(**C$_{60}$**)$_m$.

Formation of $(\textbf{PA-OCH}_3)_n$•(s/m-CNT)$_m$ and $(\textbf{PA-OCH}_3)_n$•(GN)$_m$

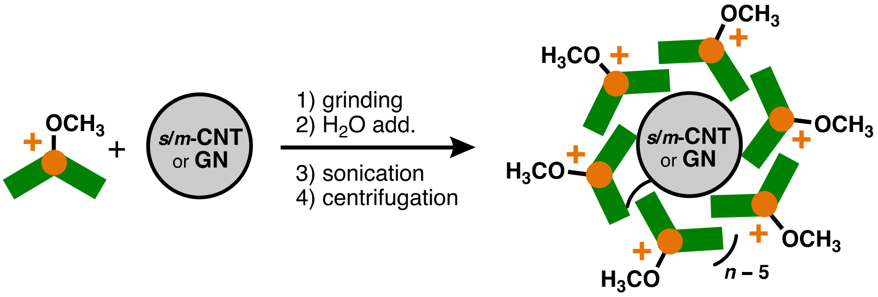

A mixture of **PA-OCH$_3$** (1.7 mg, 2.8 μmol) and ***s*-CNT** (0.4 mg; 0.7–0.9 nm thick, ≥0.7 μm long) was ground for 3 min by using an agate mortar and pestle. After addition of H_2O (2.8 mL), the suspension was sonicated (40 kHz, 150 W) for 30 min with a probe sonicator and then centrifuged (16,000*g*) for 10 min. The obtained supernatant was again centrifuged for 1 min to yield (**PA-OCH$_3$**)$_n$•(***s*-CNT**)$_m$ as a clear black solution. The solubilization of ***s*-CNT** was confirmed by UV–vis analysis. In the same way, a black aqueous solution of (**PA-OCH$_3$**)$_n$•(***m*-CNT**)$_m$ was obtained by the treatment of ***m*-CNT** (0.5 mg; 9–11 nm thick, 3-6 μm long) with **PA-OCH$_3$**. Likewise, a black aqueous solution of (**PA-OCH$_3$**)$_n$•(**GN**)$_m$ was obtained from **PA-OCH$_3$** (1.3 mg, 2.1 μmol) and **GN** (1.1 mg; 2–10 nm thick, 5 μm wide) under the same conditions. The concentration of **GN** in the solution of (**PA-OCH$_3$**)$_n$•(**GN**)$_m$ was roughly estimated as 0.03 mg mL^{-1} by weighing after the lyophilization of the isolated product and removal of **PA-OCH$_3$** via washing with CH_3OH.

Zeta-potential measurements of (PA-R)$_n$ and (PA-OCH$_3$)$_n$•(s-CNT)$_m$

A clear yellow solution (~ 1 mL) of (**PA-CH$_3$**)$_n$ in Milli-Q water (0.5 mM based on **PA-CH$_3$**) was transferred into a measurement cell and subjected to zeta-potential measurement (rt, number of scans per measurement: 100 (fixed), absorbance: 0.3 (colored sample), refractive index: 1.461). All measurements were performed in triplicate and the given values are based on the average. The machine was allowed to equilibrate for 30 min prior to measurement. In the same way, zeta-potentials of (**PA-OCH$_3$**)$_n$, (**PA-OH**)$_n$, and (**PA-Im**)$_n$ (0.5 mM based on **PA-R**) were determined. The zeta-potential of the clear colorless solution of (**AA**)$_n$ was measured at a concentration of 1.0 mM based on **AA**, considering the cmc of (**AA**)$_n$ (~ 1 mM), and an absorbance setting of 0.001 (colorless sample). In a similar way, the zeta-potential was obtained for host-guest composite (**PA-OCH$_3$**)$_n$•(***s*-CNT**)$_m$ (0.5 mM based on **PA-OCH$_3$**).

Table S1. Zeta-potentials of (**PA-R**)$_n$ and (**AA**)$_n$.

aromatic micelle	raw data [mV]	average [mV]
(**PA-CH$_3$**)$_n$	7.04, 6.20, 8.69	7.3
(**PA-OCH$_3$**)$_n$	26.3, 16.8, 13.2	18.8
(**PA-OH**)$_n$	26.9, 17.2, 16.7	20.3
(**PA-Im**)$_n$	37.0, 41.8, 46.3	41.7
(**AA**)$_n$	49.0, 49.8, 47.9	48.9

Table S2. Zeta-potential of (**PA-OCH$_3$**)$_n$•(***s*-CNT**)$_m$.

host-guest composite	raw data [mV]	average [mV]
(**PA-OCH$_3$**)$_n$•(***s*-CNT**)$_m$	42.1, 44.0, 43.6	43.2

Zeta-potential measurements of (PA-OCH$_3$ or PA-Im)$_n$•(C$_{60}$)$_m$ under neutral/acidic conditions

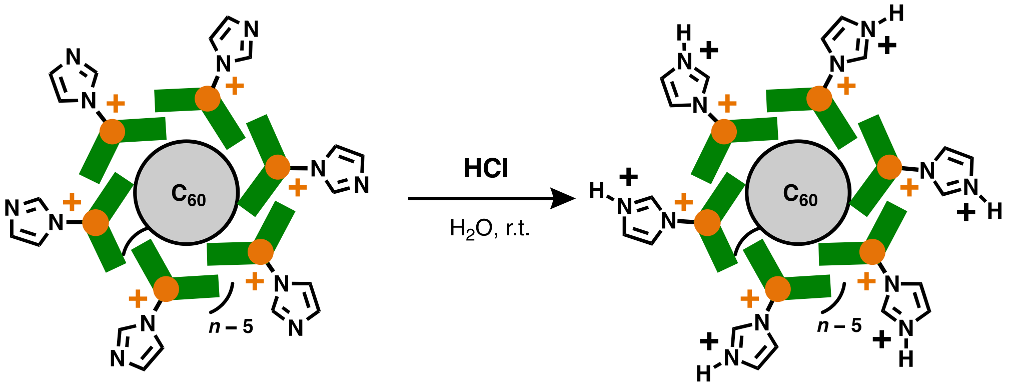

To determine the pH-responsiveness of (**PA-Im**)$_n$•(**C$_{60}$**)$_m$, a 1.0 M aqueous HCl solution (10 µL, 9 equiv based on **PA-Im**) was added to an aqueous solution (2.3 mL; pH 6.7) of (**PA-Im**)$_n$•(**C$_{60}$**)$_m$ (0.5 mM based on **PA-Im**). After mild agitation, the mixture (pH 2.8) was subjected to zeta-potential analysis. The stability of (**PA-Im**)$_n$•(**C$_{60}$**)$_m$ after HCl addition was confirmed by UV–vis measurement. In the same way, **(PA-OCH$_3$)$_n$•(C$_{60}$)$_m$** was treated with HCl and subjected to zeta-potential analysis.

Table S3: Zeta-potentials of (**PA-OCH$_3$** or **PA-Im**)$_n$•(C_{60})$_m$ under neutral/acidic conditions.

host-guest composite	raw data [mV]	average [mV]
(**PA-OCH$_3$**)$_n$•(**C_{60}**)$_m$	48.5, 45.7, 46.0	46.7
(**PA-OCH$_3$**)$_n$•(**C_{60}**)$_m$ + HCl	37.4, 48.0, 57.8	47.7
(**PA-Im**)$_n$•(**C_{60}**)$_m$	49.2, 53.4, 54.7	52.8
(**PA-Im**)$_n$•(**C_{60}**)$_m$ + HCl	58.0, 66.3, 56.6	60.3

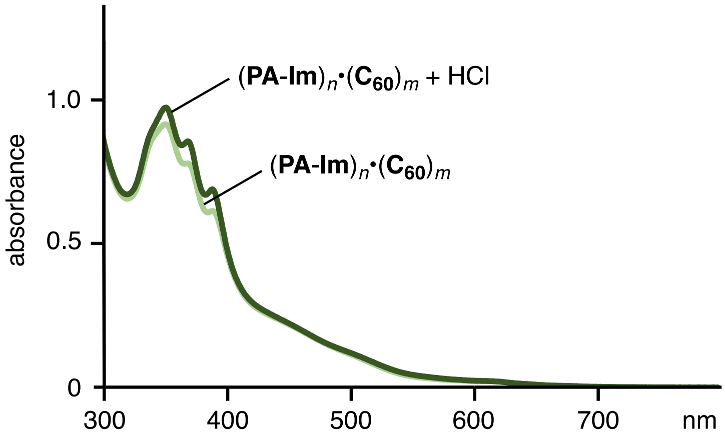

Figure S39. UV–vis spectra (H_2O, rt, 0.5 mM based on **PA-Im**) of (**PA-Im**)$_n$•(**C_{60}**)$_m$ before and after addition of HCl (9 equiv based on **PA-Im**).

Water-solubilization and deposition of g-C_3N_4

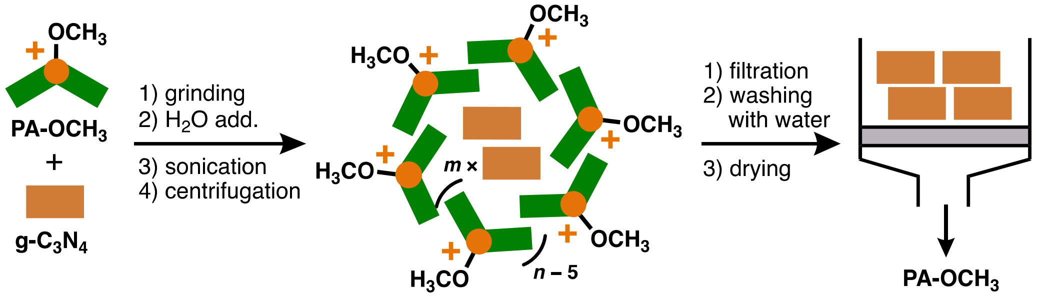

A mixture of **PA-OCH$_3$** (2.7 mg, 3.75 μmol) and **g-C_3N_4** (2.3 mg) was ground for 3 min by using an agate mortar and pestle. After addition of H_2O (4.4 mL), the suspension was sonicated (40 kHz, 150 W) for 30 min with a probe sonicator and then centrifuged (16,000*g*) for 10 min. The obtained supernatant was again centrifuged for 3 min to yield (**PA-OCH$_3$**)$_n$•(**g-C_3N_4**)$_m$ as a clear yellow solution. The solubilization of **g-C_3N_4** was confirmed by UV–vis and AFM analyses. For AFM analysis, the H_2O solution (0.25 mL) of (**PA-OCH$_3$**)$_n$•(**g-C_3N_4**)$_m$ was diluted by the addition of water (0.25 mL) and a drop of this solution (3 μL, 0.1 mM) was cast onto mica as an aerosol using a hand air blower.

After drying under a gentle air stream, the resultant mica surface was scanned in AFM tapping mode. Next, this sample on mica was washed with CH_3OH (5 × 10 μL) to remove **PA-OCH$_3$**, completely dried at rt, and then subjected again to AFM analysis.

For deposition of dissolved **g-C$_3$N$_4$**, the H_2O solution (2.0 mL) of (**PA-OCH$_3$**)$_n$•(**g-C$_3$N$_4$**)$_m$ was diluted by the addition of water (2.0 mL). The diluted aqueous solution was vacuum filtered through a mixed cellulose ester (MCE) filter (50 nm pore size), wetted with H_2O.[S4] The deposited yellow **g-C$_3$N$_4$** film was washed with water (6 mL) to remove **PA-OCH$_3$**. After drying under a stream of air, the MCE-deposited film was annealed at 200 °C for 2 h under air using a heating plate. The stability of **g-C$_3$N$_4$** toward the applied grinding–sonication protocol without **PA-OCH$_3$** was confirmed via FTIR analysis. In a similar way, ***m*-CNT** was deposited onto MCE using an aqueous solution of (**PA-OCH$_3$**)$_n$•(***m*-CNT**)$_m$. The deposited ***m*-CNT** film was subsequently washed with acetone (4 × 2 mL) to remove MCE, yielding a free-standing ***m*-CNT** film ($d \approx 0.5$ cm). The film was set afloat on acetone and transferred to a silicon substrate.

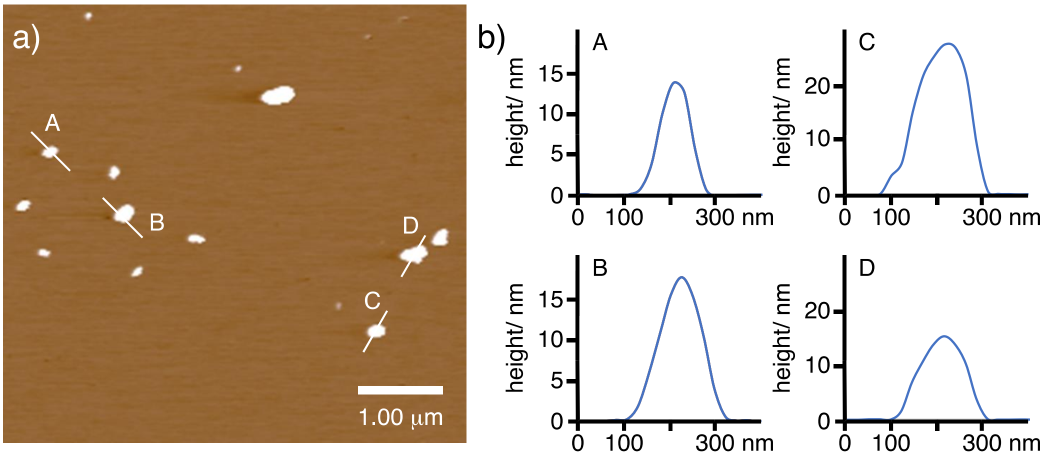

Figure S40. a) AFM image (mica, dry) of (**PA-OCH$_3$**)$_n$•(**g-C$_3$N$_4$**)$_m$ and b) the size profiles of the selected sections.

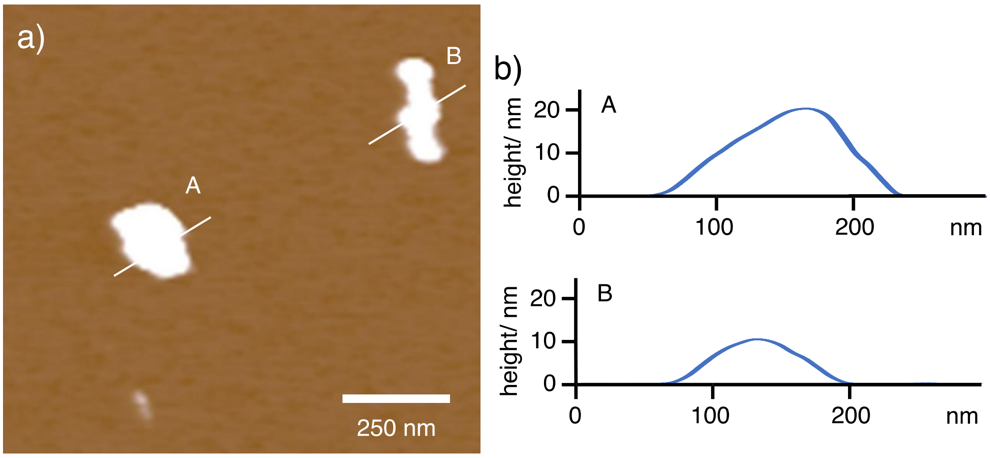

Figure S41. a) AFM image (mica, dry) of **($PA-OCH_3$)$_n$•($g-C_3N_4$)$_m$** after washing with CH_3OH and b) the size profiles of the selected sections.

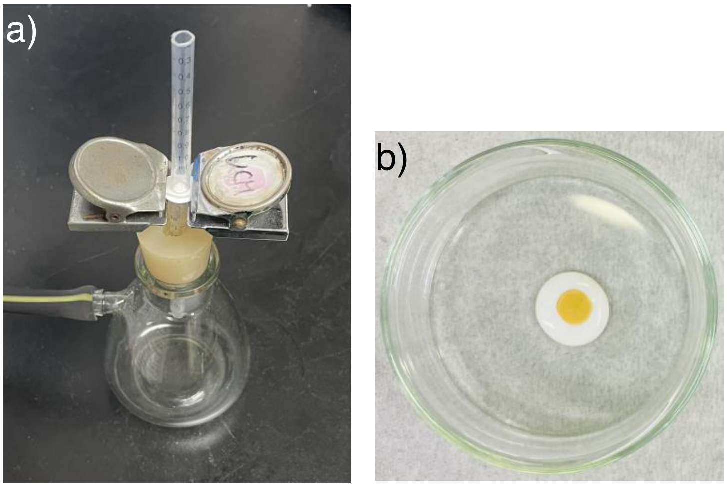

Figure S42. Photographs of a) the filtration setup and b) **$g-C_3N_4$** deposited on MCE after annealing.

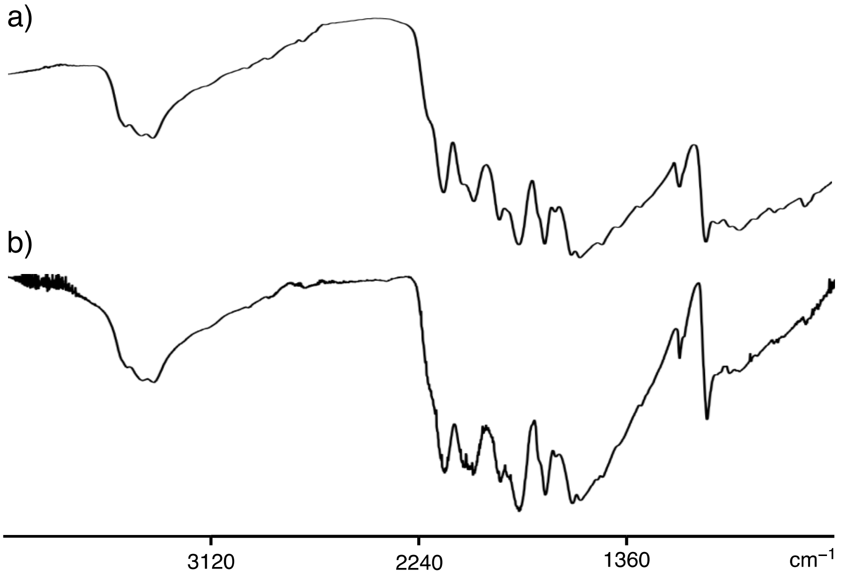

Figure S43. FTIR spectra (ATR, rt) of **g-C_3N_4** a) before and b) after grinding (3 min) and sonication (30 min) in water.

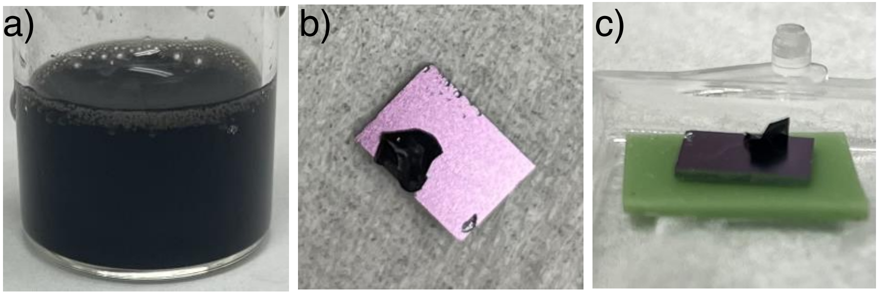

Figure S44. Photographs of a) aqueous (**PA-OCH_3**)$_n$•(***m*-CNT**)$_m$ and an ***m*-CNT** film on a silicon substrate b) directly after transfer and c) after complete drying.

Insolubility of nonionic AA'

To **AA'** (2.1 mg, 2.7 µmol) was added D_2O (2.7 mL) and the suspension was bath-sonicated for 3 min, and subsequently heated at ~ 100 °C.[4] The obtained suspension was subjected to ^1H NMR analysis, confirming the insolubility of **AA'** in water.

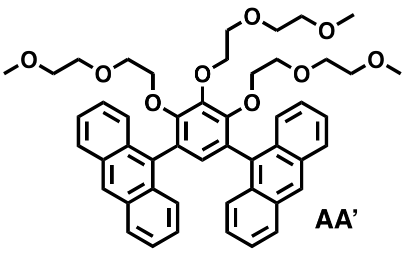

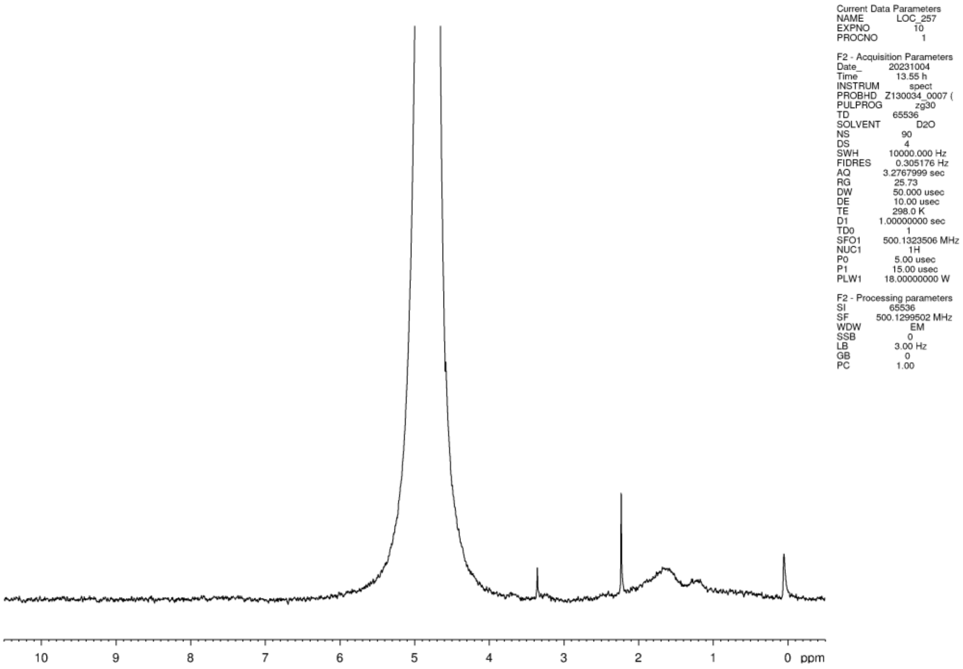

Figure S45. ^{1}H NMR spectrum (500 MHz, D_2O, rt, 1.0 mM based on **AA'**) of a suspension of **AA'**.

Formation of (PA-CH_3)$_n$•(DCM)$_m$

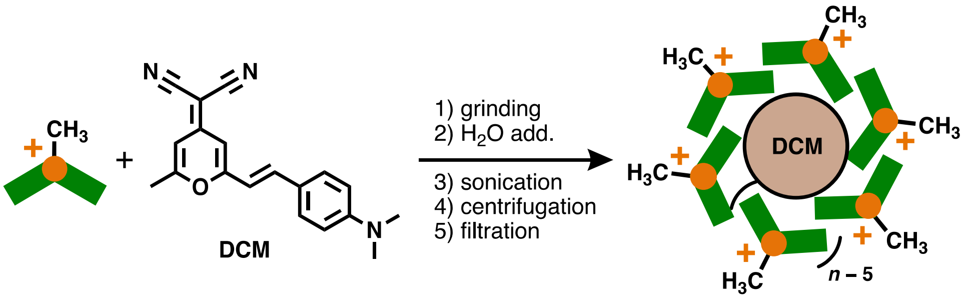

A mixture of **PA-CH_3** (1.3 mg, 2.7 μmol) and **DCM** (0.2 mg, 1.6 μmol) was ground for 5 min by using an agate mortar and pestle. After addition of H_2O (2.7 mL), the suspension was sonicated (40 kHz, 150 W) for 10 min with a probe sonicator, centrifuged (16,000*g*) for 10 min, and then filtrated by a membrane filter (pore size: 200 nm) to give a clear red solution of (**PA-CH_3**)$_n$•(**DCM**)$_m$. The solubilization of **DCM** was confirmed by UV–vis analysis.

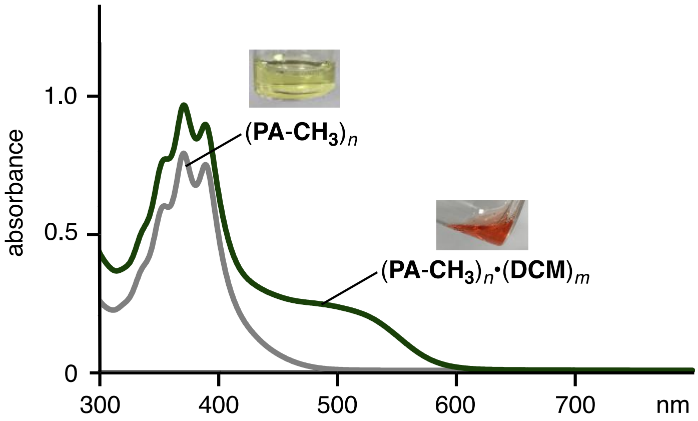

Figure S46. UV–vis spectra (H_2O, rt, 0.9 mM based on **PA-CH$_3$**) of (**PA-CH$_3$**)$_n$•(**DCM**)$_m$ and (**PA-CH$_3$**)$_n$.

Interactions of (PA-Im)$_n$ with PdCl$_2$(CH$_3$CN)$_2$

To a D_2O solution (1.5 mL) of (**PA-Im**)$_n$ (1.0 mM based on **PA-Im**) was added water-insoluble PdCl$_2$(CH$_3$CN)$_2$ (0.2 mg, 0.7 μmol) and the mixture was stirred at rt for 20 min. The resulting suspension was centrifuged and the supernatant was subjected to ^{1}H NMR analysis. Interactions between (**PA-Im**)$_n$ and Pd(II) ions were indicated by significant signal broadening. In contrast, addition of PdCl$_2$(CH$_3$CN)$_2$ to (**PA-OCH$_3$**)$_n$ led to no similar change in the ^{1}H NMR spectrum under the same conditions, indicating the importance of the imidazole units for the interactions with Pd(II) ions. A similar change in the ^{1}H NMR spectrum upon addition of PdCl$_2$(CH$_3$CN)$_2$ was observed using (**PA-Im**)$_n$•(**C$_{60}$**)$_m$.

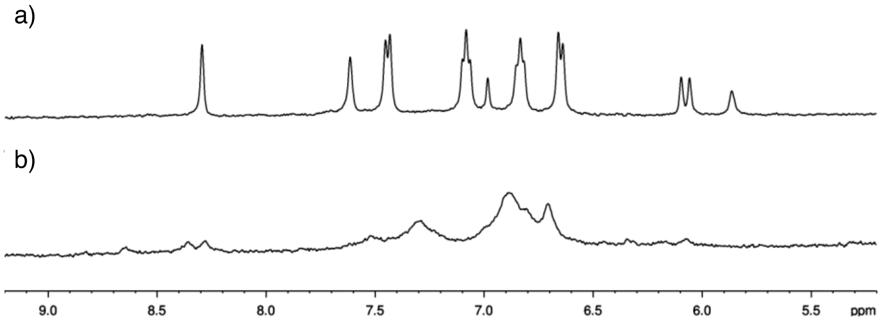

Figure S47. ^{1}H NMR spectra (400 MHz, D_2O, rt, 1.0 mM based on **PA-Im**) of a) (**PA-Im**)$_n$ and b) (**PA-Im**)$_n$ after addition of PdCl$_2$(CH$_3$CN)$_2$ (0.5 equiv) after 20 min.

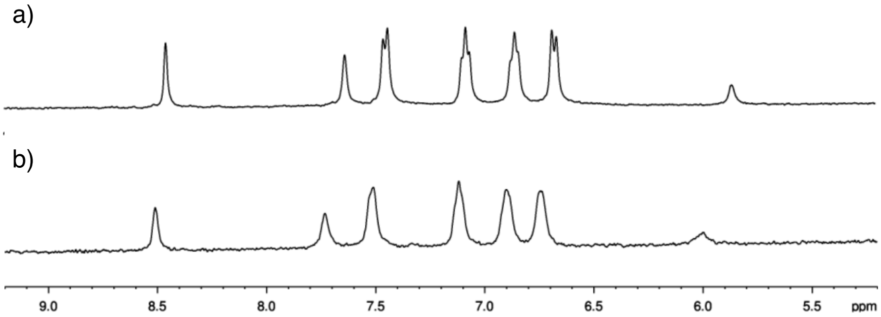

Figure S48. ^{1}H NMR spectra (400 MHz, D_2O, rt, 1.0 mM based on **PA-OCH$_3$**) of a) (**PA-OCH$_3$**)$_n$ and b) (**PA-OCH$_3$**)$_n$ after addition of $PdCl_2(CH_3CN)_2$ (0.5 equiv) after 20 min.
